# Deciphering Key microRNA Regulated Pathways in Tissue-Engineered Blood Vessels: Implications for Vascular Scaffold Production

**DOI:** 10.3390/ijms25126762

**Published:** 2024-06-20

**Authors:** Lenize da Silva Rodrigues, Tainara Francini Felix, Iael Weissberg Minutentag, Patricia Pintor Reis, Matheus Bertanha

**Affiliations:** 1 Department of Surgery and Orthopedics, Botucatu Medical School, São Paulo State University (UNESP), Botucatu 18618-687, SP, Brazil; lenizerodrigues@gmail.com; 2 Applied Biotechnology Laboratory, Clinical Hospital of Botucatu Medical School, São Paulo State University (UNESP), Botucatu 18618-687, SP, Brazil; 3 Experimental Research Unit, Botucatu Medical School, São Paulo State University (UNESP), Botucatu 18618-687, SP, Brazil; tainara.felix@unesp.br (T.F.F.); iael.weissberg@unesp.br (I.W.M.); patricia.reis@unesp.br (P.P.R.)

**Keywords:** peripheral arterial disease, stem cells, tissue transplant-based therapy

## Abstract

MicroRNAs (miRNAs) are non-coding RNAs involved in the regulation of gene expression associated with cell differentiation, proliferation, adhesion, and important biological functions such as inflammation. miRNAs play roles associated with the pathogenesis of chronic degenerative disorders including cardiovascular diseases. Understanding the influence of miRNAs and their target genes can effectively streamline the identification of key biologically active pathways that are important in the development of vascular grafts through the tissue engineering of blood vessels. To determine miRNA expression levels and identify miRNA target genes and pathways with biological roles in scaffolds that have been repopulated with adipose-derived stem cells (ASCs) generated through tissue engineering for the construction of blood vessels. miRNA quantification assays were performed in triplicate to determine miRNA expression in a total of 20 samples: five controls (natural inferior vena cava), five scaffolds recellularized with ASCs and differentiated into the endothelium (luminal layer), five samples of complete scaffolds seeded with ASCs differentiated into the endothelium (luminal layer) and smooth muscle (extraluminal layer), and five samples of ASC without cell differentiation. Several differentially expressed miRNAs were identified and predicted to modulate target genes with roles in key pathways associated with angiogenesis, vascular system control, and endothelial and smooth muscle regulation, including migration, proliferation, and growth. These findings underscore the involvement of these pathways in the regulatory mechanisms that are essential for vascular scaffold production through tissue engineering. Our research contributes to the knowledge of miRNA-regulated mechanisms, which may impact the design of vascular substitutes, and provide valuable insights for enhancing clinical practice. The molecular pathways regulated by miRNAs in tissue engineering of blood vessels (TEBV) allowed us to elucidate the main phenomena involved in cellular differentiation to constitute a blood vessel, with the main pathways being essential for angiogenesis, cellular differentiation, and differentiation into vascular smooth muscle.

## 1. Introduction

Peripheral Arterial Obstructive Disease (PAOD) is one of the most prevalent cardiovascular diseases (CVDs), leading to physical limitations and worsening of patient quality of life [1,2]. PAOD affects 200 million people with CVD worldwide and is highly related to age, being particularly prevalent among individuals aged 60–70 years old [3,4,5]. The surgical management of atherosclerotic obstructive disease, encompassing both coronary and peripheral conditions, may lead to interventions involving the establishment of grafts or bypass procedures. In cases where the autologous great saphenous vein is unavailable, synthetic arterial substitutes such as Dacron^®^ or polytetrafluoroethylene (PTFE) prostheses may be used. Nevertheless, the employment of these synthetic substitutes must be prudently considered, as their unrestricted use can potentially give rise to new lesions or infections, consequently leading to elevated treatment failure rates [1,5,6,7,8,9,10]. In this context, a promising and compelling alternative lies in future utilization of vascular grafts produced through tissue engineering of blood vessels (TEBV). These grafts are constructed using decellularized bioscaffolds and autologous stem cells, facilitating neovascularization. This approach has demonstrated its potential as an abundant and immunologically compatible source, offering proper resistance, remodeling capabilities, and innate self-repair capacity [11].

The formation of new functional blood vessels from a bioscaffold by TEBV is considered a delicate balance that involves the maintenance of cellular properties, such as migration, proliferation, and differentiation, as well as gene regulatory pathways. Notably, miRNAs, as potent gene expression regulators, are thought to contribute to normal integration of new blood vessels into the vascular system [12].

In the context of the vascular system, physiological balance occurs through a tightly regulated process of angiogenesis, and a disruption in this regulatory process may represent a risk factor for the development of cardiovascular diseases [13,14,15,16,17]. Angiogenesis is responsible for the control of normal and pathological physiological processes. It regulates several molecular signaling events and ensures interactions between various cell types. The main steps in angiogenesis involve activation of endothelial cells (EC), enzymatic degradation of the capillary basement membrane, proliferation of EC, directed migration, fusion of vessels, reduction and stabilization of pericytes, and formation of the capillary network [18,19,20,21]. This process occurs through angiogenic regulators and signaling molecules, including Vascular Endothelial Growth Factor-A (VEGF-A), Fibroblast Growth Factor (FGF), Epidermal Growth Factor (EGF), and Interferon, Matrix Metalloproteinase-1/9 (MMP-1/9). The mechanism is regulated through angiogenic stimulators such as Angiogenin, Angiopoietin, transforming growth factor α/β (TGF α/β), and angiogenic inhibitors such as Angioarrestin, Interleukin 12, fibronectin fragment, and angiostatin [18,19,20,21]. Angiogenesis is also modulated by miRNAs [22,23]. miRNAs are small non-coding RNAs with potent roles in gene expression regulation associated with various biological processes, including cell development, differentiation, and proliferation [24].

Thus, miRNAs are implicated in pathophysiological processes associated with both healthy and diseased individuals. Such processes include cell adhesion, proliferation, and lipid uptake, as well as the function of inflammatory mediators. miRNAs are associated with atherosclerosis and may represent new therapeutic targets for intervention [25]. These changes lead to increased cell proliferation and have been associated with the pathogenesis of different types of cardiovascular diseases, including vascular restenosis following angioplasty [26,27].

Certain miRNAs, such as *miR-126*, *miR-221/222*, *miR-17-92 cluster*, *miR-93*, *let-7f*, and *miR-214*, have been reported to modulate the vascular endothelium response to angiogenic stimuli [28]. The term “angiomiRNAs” or “angiomiRs” has been proposed to refer to miRNAs that regulate angiogenesis [29,30,31,32,33,34,35,36]. Several miRNAs have been found to have ectopic expression exclusively in EC, with lineage-specific expression in endothelial lineage cells [37]. These miRNAs affect angiogenesis by interacting with various key molecules in different cell types. Several miRNAs play distinct roles in angiogenesis. Pro-angiogenic miRNAs, such as *miR-126*, promote VEGF-dependent AKT and ERK signaling by derepressing p85. *miR-210* controls capillary-like formation and EC chemotaxis in response to VEGF. In addition, *miR-10b* and *miR-9b* regulate the increase in EC proliferation induced by VEGF, while *miR-17-92* promotes endothelial cell division and migration. In contrast, *miR-24* inhibits angiogenesis, *miR-221* and *miR-222* inhibit EC migration and proliferation, and *miR-15b* induces apoptosis. Additionally, *miR-192* negatively regulates the global angiogenic pathway.

The objective of our study was to determine miRNA expression of decellularized scaffolds that were recellularized with stem cells differentiated into the endothelium and smooth muscle. This approach allowed us to gain a deeper understanding of normal miRNA-regulated pathways potentially associated with the embryological process of angiogenesis. By identifying genetic pathways involved, we aimed to identify the essential biomolecular characteristics necessary for TEBV. Ensuring that these characteristics align with the criteria for seamless integration and optimal vessel function is crucial for advancements in regenerative medicine and tissue replacement therapies.

## 2. Results

### 2.1. Significantly Deregulated miRNAs, miRNA Target Prediction, and Pathway Analysis

#### 2.1.1. Endothelialized Scaffold versus Controls (Natural Veins): miRNA Target Genes Are Mainly Related to the MAPK Pathway

A total of 605 miRNAs were significantly deregulated (FC ≥ 2 and *p* ≤ 0.05) in the endothelialized scaffold compared to controls (natural vein). Bonferroni correction was applied to these data, and we identified 185 miRNAs with *p* ≤ 0.00002.

We selected 15 miRNAs, based on FC and Bonferroni-corrected *p*-values (Table 1). They were used to predict target genes in the microRNA Data Integration Portal (miRDIP) database. A total of 3292 target genes of these 15 miRNAs were identified. Next, we sought to identify which genes were regulated by more than one miRNA. This analysis revealed that 915 out of the 3292 target genes were regulated by at least five of the fifteen miRNAs (Supplemental Table S1). Such genes were selected for further pathway analysis. We found that this strategy allowed the identification of genes into biologically relevant networks.

Pathway analysis was performed using EnrichR [https://maayanlab.cloud/Enrichr/] (accessed on 1 April 2024), a tool that provides results from various databases, offering several levels of interpretation, including Molecular Pathways, Biological Function, among others [38,39,40].

**Figure 1:**
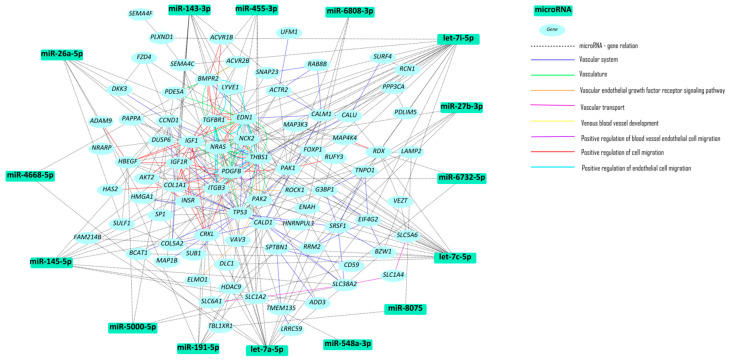
Network of molecular pathways of the endothelialized scaffold versus control.

Our results showed that the most statistically significant pathway was the MAPK pathway, identified in five out of the six major databases (Jensen Tissue, GO Biological Process 2021, GO Molecular Function, KEGG 2021 Human, and WikiPathway 2021 Human). The MAPK/ERK (also known as the Ras-Raf-MEK-ERK pathway) has signaling activity that begins with the stimulation of a cell membrane receptor and ends with the activation of gene transcription in the cell nucleus. This pathway plays a crucial role in various key cellular processes, including cell division, and is of significant interest in the context of vascular diseases (Figure 1).

#### 2.1.2. Endothelialized Scaffold with Smooth Muscle versus Control (Natural Vein): miRNAs Target Genes Are Mainly Related to EGF/EGFR, BMPR2 and VEGFA/VEGFR Regulatory Pathways

Bonferroni correction identified 18 statistically significantly differentially expressed miRNAs (eighteen over-expressed and five under-expressed in endothelialized scaffold with smooth muscle versus control). Among the eighteen miRNAs, three (miR-3135b, miR-378g, and miR-4433b-3p) did not show predicted target genes with the minimum score “very high” in the prediction analysis (Table 2). For the remaining 18 miRNAs, target prediction analysis showed a total of 5099 genes (Supplemental Table S2). Among the 5099 target genes, 1336 were regulated by at least five of the eighteen miRNAs. Therefore, we used the list of 1336 genes for further pathway analysis in EnrichR.

**Figure 2:**
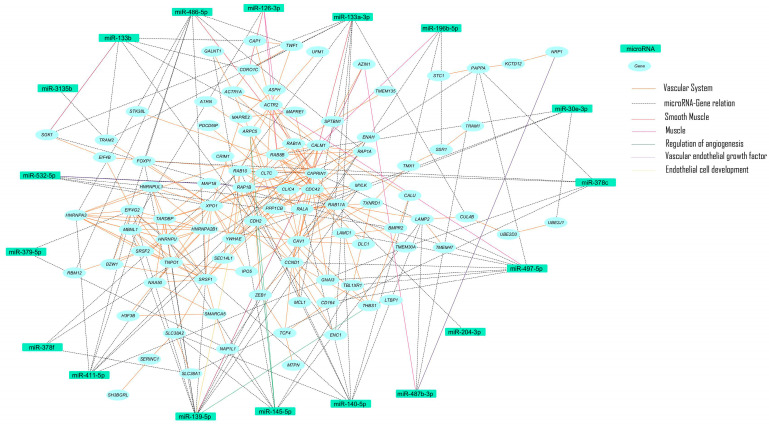
Network of molecular pathways of the scaffold endothelialized with smooth muscle versus control.

Molecular pathways identified included: EGF/EGFR; Bone Morphogenetic Protein Receptor Type II (BMPR2); VEGFA/VEGFR2; vascular system; endothelial differentiation; vascular endothelial; vascular smooth muscle cell—embryonic stem cell; mesenchymal stem cell—muscle; and smooth muscle cell—blood vessel (Figure 2).

Our findings showed pathways important to angiogenesis, which are extremely relevant to the pathophysiology of the vascular system and may be affected in vascular diseases.

### 2.2. Scaffold Recellularized with Differentiated ASC versus Undifferentiated Stem Cell

#### 2.2.1. Endothelialized Scaffolds versus Undifferentiated Cells

**Figure 3:**
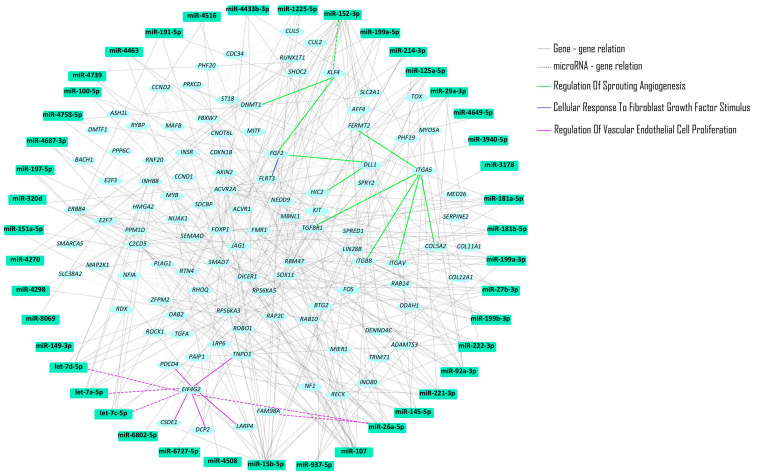
Molecular pathway network of endothelial cells versus undifferentiated MSC.

miRNA expression analysis identified 57 miRNAs with FC ≥ 2 and Bonferroni-corrected *p* ≤ 0.00002 in the endothelialized scaffold compared to the undifferentiated ASC (control) (Table 3). The 57 identified miRNAs were then used for target prediction analysis in miRDIP. This analysis resulted in 15,100 target genes with 368 of them being regulated by at least five miRNAs (Supplemental Table S3). Figure 3 shows a protein–protein–miRNA interaction network for the 368 genes regulated by the 57miRNAs.

Molecular pathways identified included: EGF/EGFR; VEGFA/VEGFR2; vascular system; endothelial differentiation; vascular endothelial; mesenchymal stem cell; vascular endothelial growth factor receptor or signaling pathway; and positive regulation of endothelial cell migration (Figure 3).

#### 2.2.2. Complete Scaffold (New Blood Vessel with Endothelium and Smooth Muscle) versus Undifferentiated Cells

**Figure 4:**
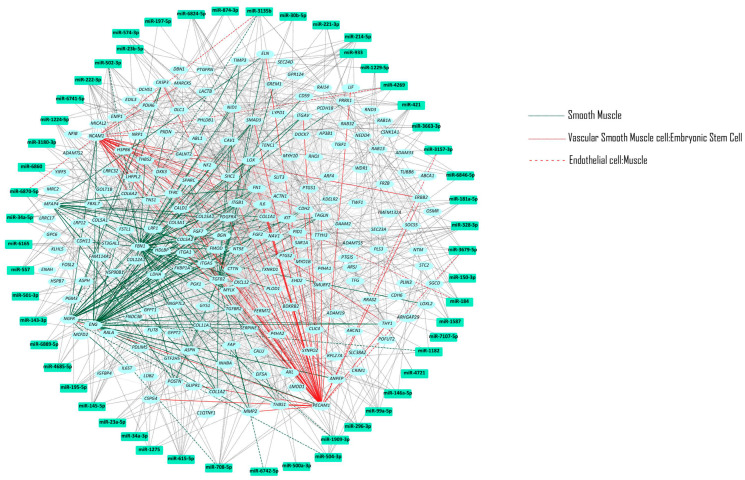
Molecular pathway network of smooth muscle cells versus undifferentiated MSCs.

miRNA expression analysis identified 52 miRNAs with FC ≥ 2 and *p* ≤ 0.0008 in the complete scaffold compared to undifferentiated cells (Table 4). These 52 miRNAs were then used for target prediction in miRDIP. We identified 10,993 target genes (Supplemental Table S4). Of these, 428 were regulated by at least five of the fifty-two miRNAs (Figure 4).

Molecular pathways identified included: EGF/EGFR; Bone Morphogenetic Protein Receptor Type II (BMPR2); VEGFA/VEGFR2; vascular system; endothelial differentiation; vascular endothelial; vascular smooth muscle cell—embryonic stem cell; mesenchymal stem cell—muscle; smooth muscle cell—blood vessel; mesenchymal stem cell; vascular endothelial growth factor receptor or signaling pathway; and positive regulation of endothelial cell migration.

## 3. Discussion

Biological scaffolds are a source of biomaterials with many advantages, such as low toxicity, low immunogenicity, good cell adhesion, and proliferation capacity. When using biological scaffolds, the native architecture is highly preserved, and the antigen content is removed during the decellularization process [41,42,43]. The use of decellularized veins as biological scaffolds for tissue engineering in blood vessels was chosen as a biocompatible, resistant, and adaptable 3D tubular structure, which had good results in cell culture in experiments previously carried out by our study team [44,45,46,47,48,49].

A better understanding of gene expression regulation pathways and cell cycle/proliferation in TEBV is important for the evaluation of possible decreased gene expression and the biomolecular behavior involved. This is applied both in regenerative processes and the development of cardiovascular diseases [41,42,43].

miRNAs have a specific pattern of expression and gene regulation in embryonic stem cells, with the most highly expressed being *miR-17-92*, *miR-290-295*, *miR-302*, and *miR-106b-25*, which regulate cell cycle phases and cell differentiation [43,50,51,52,53,54,55]. We compared the recellularized scaffolds with both EC and the completely recellularized new blood vessels with endothelial and smooth muscle cells to what we consider the natural control of the vein, in addition to the comparison with cultured ASCs. miRNA analysis demonstrated a significant variation in upregulated genes related to the natural processes of formation and development, embryonic and adult, of the constituent cells of the cardiovascular system, as well as some pathways involved in disease processes.

miRNAs play a fundamental role in the regulatory pathways that controls the differentiation of human cells toward the endothelial lineage, blood vessel formation, and self-renewal of embryonic cells [56]. The importance of controlling cell regulation through miRNA expression in the development of the cardiovascular system has been increasingly studied, demonstrating that loss of this function can compromise the differentiation and development of cardiac cells and blood vessel formation, leading to agenesis of the cardiovascular system [57]. Some studies have focused on the role of miRNAs in regulating vasculogenesis in vascular diseases; however, most have focused on regulating cell phenotypes in their migration and proliferation. Nevertheless, other studies have highlighted the individual roles of miRNAs in the differentiation and regulation of specific genes in EC [58,59,60,61].

Therefore, it is important to analyze the expression of miRNAs that regulate angiogenesis and individual genes in specific EC that directly affect the development of the cardiovascular system, which are still being explored. In this study, we aimed to use miRNA gene expression analysis in neovasculature produced by TEBV to confirm the presence of specific genes in the recellularization of decellularized vessels and specific genetic pathways during the vascular induction process. We identified various highly expressed and active pathways that provide more information about the evolution of TEBV and its underlying mechanisms [62].

In the intricate landscape of molecular biology and tissue engineering, this study represents a significant leap forward in our journey to understand the fine-tuned intricacies of miRNA regulation and gene expression within the realm of TEBV. As we navigate the complexities of this molecular orchestra, we uncover a fascinating narrative of how miRNAs serve as conductors, orchestrating the symphony of differentiation in undifferentiated human cells towards the noble lineage of the endothelium, and ultimately, the construction of intricate vascular networks.

The heart of our research lies in the influence of miRNAs on the delicate balance of gene expression during recellularization of decellularized vessels. These small RNA molecules act as mediators, guiding cells along the intricate paths of vascular rebirth. These findings highlight the importance of miRNA-mediated regulation in steering the developmental course of the cardiovascular system, one of the most critical systems in the human body.

As we delve deeper into this molecular tapestry, we unravel a complex network of gene expression and regulatory pathways. This intricate architecture underpins the recellularization process and forms the cornerstone of our understanding of TEBV. Beyond TEBV, these insights hold promise for a wide array of regenerative and therapeutic applications, potentially reshaping the future of cardiovascular health and tissue engineering.

Our findings suggest that new blood vessels are still in the process of formation, as they exhibit highly active cell division-related pathways. Additionally, the TGFβ signaling pathway, found in two of the six databases, plays diverse roles in both embryogenesis and adult individuals, including the regulation of extracellular matrix (ECM) neogenesis in mesodermal cells. The Jensen Tissue database includes regulatory pathways related to angiopoietin, which belongs to the vascular growth factor family and is important for both embryonic and postnatal angiogenesis. Angiopoietin signaling is more directly associated with angiogenesis, the process by which new arteries and veins form from preexisting blood vessels. Signaling pathways related to pluripotency were identified in three of the six major databases (Jensen Tissue, GO Biological Process 2021, and GO Molecular Function) and are commonly associated with cell growth and mitosis. Among these genes, we considered that functions related to cell proliferation and differentiation were the most important in the context of this study.

Many of the identified miRNAs with increased expression showed very high fold-change values (up to 1000×). Similar results have been reported for situations in which miRNA expression is induced by a specific treatment.

Moreover, our exploration of miRNA profiles offers a treasure trove of insights into the genes and pathways that are actively engaged in the creation and maturation of the cardiovascular system. This newfound knowledge empowers us to not only grasp the intricacies of angiogenesis but also to envision innovative approaches for regenerative medicine. The intricate ballet of genes and miRNAs discovered in this study provides a rich source of information that can guide future endeavors aimed at unlocking the potential of miRNAs in cardiovascular health and TEBV.

## 4. Methods and Materials

### 4.1. Animal Handling Conditions and Tissue Acquisition

Animals were handled in accordance with the National Institute of Health (NIH) Guidelines for the Care and Use of Laboratory Animals (NIH 2011), with approval from the local Committee on Animal Research Ethics (CARE) under registration #1279/2018. Ten adult New Zealand rabbits were used, all non-pregnant females weighing between 2.5 and 3.5 kg. IVCs and 2 g of adipose tissue (AT) were obtained from these animals to generate the cell bank (Supplemental File S1, Supplemental Figures S1.1 and S1.2). Animals were housed under controlled conditions and fed a standard pellet diet with water ad libitum. Before the procedures to obtain the AT and IVC, animals were given an intravenous lethal dose of the anesthetic pentobarbital (150 mg/kg), according to the “Guidelines for the Practice of Euthanasia of CONCEA”.

### 4.2. Samples

The samples used for miRNA analysis were obtained from 10 scaffolds (Supplemental File S2, and Supplemental Figure S2). These scaffolds were reseeded with adipose tissue-derived mesenchymal stem cells (ASC), differentiated into EC for the luminal layer (Supplemental File S3, and Supplemental Figure S3) and smooth muscle cells for the extraluminal layer (Supplemental File S4, and Supplemental Figure S4), and cultured for 8 weeks in DMEM medium with growth factors for each lineage. Among the ten scaffolds, five underwent cellular differentiation, whereas the remaining five did not. The control samples included fresh inferior vena cava tissue and undifferentiated ASC samples. All samples were kept in simple DMEM for 24 h to remove fetal bovine serum protein and washed with PBS before miRNA expression analysis.

### 4.3. miRNA Expression Analysis

Vein fragments, recellularized scaffolds, and ASC from cell culture were collected and immediately frozen before RNA extraction and miRNA expression analysis.

#### 4.3.1. RNA Extraction

Total RNA was isolated from vascular scaffolds and fresh frozen vein tissues using the standard Trizol/chloroform protocol [63,64]

#### 4.3.2. Quantification Analysis of miRNA Expression

miRNA quantification analysis was performed using the GeneChip^®^ microRNA 4.0 array (Thermo Fisher Scientific, Waltham, MA, USA), which contains probes representing all 2588 miRNAs annotated on miRBase version 21 (https://mirbase.org/ accessed on 1 April 2024). The procedures were performed in accordance with manufacturer’s protocol. In summary, RNA samples were first subjected to 3′-end poly-A tailing, followed by a second step of ligation and biotinylation of the 3′-poly A tail of RNA. The biotin-labeled RNA from each sample was then hybridized to the GeneChip^®^ miRNA 4.0 array cartridge and detection was carried out using the avidin–streptavidin–phycoerythrin (PE) conjugate, which binds strongly to biotin-labeled RNA. The miRNA matrix cartridges were then placed in hybridization oven trays, loaded into the hybridization oven, and incubated at 48 °C with 60 rpm rotation for 16 h. Upon hybridization, each array was filled with array holding buffer and allowed to reach room temperature before washing and staining. Washing and staining steps were performed using the appropriate fluidic script for cartridge arrays on the fluidic station. The arrays were then scanned, and the data were exported for further analysis using the Expression Console software (Affymetrix version 4.0.1) for data summarization, normalization, and quality control verification.

#### 4.3.3. miRNA Target Prediction Analysis

Differentially expressed miRNAs in scaffolds were then subjected to target prediction analysis using MicroRNA Data Integration Portal (http://ophid.utoronto.ca/mirDIP/ accessed on 1 April 2024), where the selection criterion for target gene prediction was “very high” (top 1%) scores for interaction probability.

### 4.4. Statical Analyses

Data analysis was performed using the Transcriptome Analysis Console (TAC) software version 4.0.1. And, based on FC and Bonferroni-corrected *p*-values, data were analyzed independently to identify significantly expressed normal miRNAs (FC ≥ 2 and *p* < 0.05) in each scaffold type compared to their corresponding normal venous tissues.

## 5. Conclusions

In summary, this study serves as evidence to the immense potential of miRNAs as central players in the choreography of cellular processes. miRNAs participate in the symphony of life from the earliest stages of differentiation to the intricate development of vascular structures. As we continue to decipher the complex score of molecular mechanisms, we move closer to harnessing their extraordinary power for the improvement of cardiovascular health and advancement of tissue engineering.

## Figures and Tables

**Table 1:** Analysis of miRNA expression profile (endothelium versus control).

Transcript ID (Array Design)	Fold Change	P-Val	FDR P-Val
hsa-miR-548a-3p	−20.69	6.27 × 10^−7^	2.13 × 10^−5^
hsa-miR-5000-5p	−38.47	1.19 × 10^−6^	3.64 × 10^−5^
hsa-miR-6732-5p *	−10.52	3.59 × 10^−6^	9.55 × 10^−5^
hsa-miR-8075 *	−18.44	4.25 × 10^−6^	0.0001
hsa-miR-455-3p	−8.08	7.36 × 10^−6^	0.0002
hsa-miR-6808-3p *	−19.87	8.86 × 10^−6^	0.0002
hsa-miR-4668-5p	−56.88	6.58 × 10^−6^	0.0002
hsa-miR-145-5p	11,136.11	6.37 × 10^−13^	4.23 × 10^−9^
hsa-miR-26a-5p	6540.45	2.72 × 10^−9^	3.38 × 10^−7^
hsa-miR-143-3p	5428.29	4.94 × 10^−9^	4.89 × 10^−7^
hsa-let-7c-5p	3311.85	3.06 × 10^−10^	1.20 × 10^−7^
hsa-let-7a-5p	3039.95	9.65 × 10^−9^	7.90 × 10^−7^
hsa-let-7i-5p	1148.94	1.18 × 10^−10^	1.02 × 10^−7^
hsa-miR-27b-3p	1134.54	1.86 × 10^−9^	2.46 × 10^−7^
hsa-miR-191-5p	1096.44	6.79 × 10^−10^	1.69 × 10^−7^

Bonferroni correction (0.05 divided by 2.578 mature miRNAs included in the array) = *p*-value for significance = 0.00002. * miRNAs in bold were not found in the prediction databases (therefore, there are no gene targets identified for these miRNAs).

**Table 2:** Analysis of miRNA expression profile (complete versus control).

Transcript ID (Array Design)	Fold Change	P-Val	FDR P-Val
hsa-miR-126-3p	351.93	3.83 × 10^−10^	1.27 × 10^−6^
hsa-miR-145-5p	97.26	2.95 × 10^−10^	1.27 × 10^−6^
hsa-miR-497-5p	66.25	7.05 × 10^−10^	1.33 × 10^−6^
hsa-miR-133a-3p	48.98	8.03 × 10^−10^	1.33 × 10^−6^
hsa-miR-379-5p	16.64	4.75 × 10^−9^	6.13 × 10^−6^
hsa-miR-140-5p	37.19	1.18 × 10^−8^	9.80 × 10^−6^
hsa-miR-378c	52.75	1.70 × 10^−8^	1.11 × 10^−5^
hsa-miR-133b	33.46	1.60 × 10^−8^	1.11 × 10^−5^
hsa-miR-139-5p	71.15	2.59 × 10^−8^	1.16 × 10^−5^
hsa-miR-487b-3p	17.81	2.43 × 10^−8^	1.16 × 10^−5^
hsa-miR-30e-3p	14.26	2.29 × 10^−8^	1.16 × 10^−5^
hsa-miR-378f	36.84	3.88 × 10^−8^	1.35 × 10^−5^
hsa-miR-3135b	21.51	3.64 × 10^−8^	1.35 × 10^−5^
hsa-miR-204-3p	14.79	3.67 × 10^−8^	1.35 × 10^−5^
hsa-miR-196b-5p	8.14	4.53 × 10^−8^	1.50 × 10^−5^
hsa-miR-486-5p	29.78	4.77 × 10^−8^	1.51 × 10^−5^
hsa-miR-532-5p	17.48	6.01 × 10^−8^	1.73 × 10^−5^
hsa-miR-411-5p	6.69	6.28 × 10^−8^	1.73 × 10^−5^

All miRNAs in this list were used for prediction of targets and pathways analyses to increase our chances of finding differences related to muscle cells.

**Table 3:** Expression profile analysis of positive miRNAs endothelialized scaffold versus control (undifferentiated ASC).

Transcript ID (Array Design)	Fold Change	P-Val	FDR P-Val
hsa-miR-4433b-3p	302.98	1.15 × 10^−14^	7.53 × 10^−11^
hsa-miR-4463	220.35	1.68 × 10^−14^	7.53 × 10^−11^
hsa-miR-145-5p	462.1	3.12 × 10^−14^	7.53 × 10^−11^
hsa-miR-4651	107.7	8.81 × 10^−14^	1.77 × 10^−10^
hsa-miR-6722-3p	98.59	3.51 × 10^−13^	5.53 × 10^−10^
hsa-miR-149-3p	370.76	5.51 × 10^−13^	6.16 × 10^−10^
hsa-let-7d-5p	465.96	7.14 × 10^−13^	6.16 × 10^−10^
hsa-miR-4649-5p	90.39	8.44 × 10^−13^	6.53 × 10^−10^
hsa-miR-222-3p	82.13	9.55 × 10^−13^	6.53 × 10^−10^
hsa-miR-3940-5p	204.43	1.03 × 10^−12^	6.94 × 10^−10^
hsa-miR-1227-5p	52.73	2.71 × 10^−12^	1.31 × 10^−9^
hsa-miR-4516	264.92	3.18 × 10^−12^	1.39 × 10^−9^
hsa-miR-125a-5p	333.65	4.66 × 10^−12^	1.56 × 10^−9^
hsa-miR-6125	79.91	5.45 × 10^−12^	1.69 × 10^−9^
hsa-miR-4687-3p	124.33	6.77 × 10^−12^	1.79 × 10^−9^
hsa-let-7c-5p	1577.71	7.51 × 10^−12^	1.79 × 10^−9^
hsa-miR-4270	136.41	8.77 × 10^−12^	1.79 × 10^−9^
hsa-miR-92a-3p	121.97	8.83 × 10^−12^	1.79 × 10^−9^
hsa-miR-214-3p	308.01	9.91 × 10^−12^	1.79 × 10^−9^
hsa-miR-3135b	82.04	9.99 × 10^−12^	1.79 × 10^−9^
hsa-miR-199a-3p	508.17	1.00 × 10^−11^	1.79 × 10^−9^
hsa-miR-199b-3p	508.17	1.00 × 10^−11^	1.79 × 10^−9^
hsa-miR-8089	41.9	1.37 × 10^−11^	2.11 × 10^−9^
hsa-miR-320c	154.25	1.84 × 10^−11^	2.79 × 10^−9^
hsa-miR-191-5p	231.72	2.34 × 10^−11^	3.26 × 10^−9^
hsa-miR-320d	68.02	2.54 × 10^−11^	3.26 × 10^−9^
hsa-miR-181a-5p	103.41	2.69 × 10^−11^	3.26 × 10^−9^
hsa-miR-99a-5p	72.57	2.78 × 10^−11^	3.26 × 10^−9^
hsa-miR-4508	329.47	3.79 × 10^−11^	4.33 × 10^−9^
hsa-miR-320b	146.38	4.74 × 10^−11^	5.20 × 10^−9^
hsa-miR-4739	37.43	4.97 × 10^−11^	5.22 × 10^−9^
hsa-miR-15b-5p	100.16	5.01 × 10^−11^	5.22 × 10^−9^
hsa-miR-4758-5p	30.27	7.13 × 10^−11^	6.13 × 10^−9^
hsa-let-7a-5p	1439.79	7.75 × 10^−11^	6.13 × 10^−9^
hsa-miR-221-3p	104.34	7.77 × 10^−11^	6.13 × 10^−9^
hsa-miR-6765-5p	93.94	8.26 × 10^−11^	6.40 × 10^−9^
hsa-miR-4505	50.95	8.41 × 10^−11^	6.48 × 10^−9^
hsa-miR-8069	91.17	1.00 × 10^−10^	7.63 × 10^−9^
hsa-miR-26a-5p	695.23	1.42 × 10^−10^	9.92 × 10^−9^
hsa-miR-100-5p	315.98	1.56 × 10^−10^	1.02 × 10^−8^
hsa-miR-29a-3p	67.5	1.61 × 10^−10^	1.04 × 10^−8^
hsa-miR-937-5p	85.25	1.68 × 10^−10^	1.07 × 10^−8^
hsa-miR-6802-5p	35.74	1.76 × 10^−10^	1.11 × 10^−8^
hsa-miR-151a-5p	106.49	2.32 × 10^−10^	1.40 × 10^−8^
hsa-miR-1225-5p	43.99	2.33 × 10^−10^	1.40 × 10^−8^
hsa-miR-6819-5p	19.7	2.38 × 10^−10^	1.41 × 10^−8^
hsa-miR-7847-3p	132.26	2.71 × 10^−10^	1.60 × 10^−8^
hsa-miR-6727-5p	49.14	2.92 × 10^−10^	1.70 × 10^−8^
hsa-miR-4734	29.73	3.66 × 10^−10^	2.06 × 10^−8^
hsa-miR-3178	89.65	5.52 × 10^−10^	3.07 × 10^−8^
hsa-miR-107	66.86	6.05 × 10^−10^	3.21 × 10^−8^
hsa-miR-4298	62.1	6.14 × 10^−10^	3.25 × 10^−8^
hsa-miR-152-3p	101.91	6.37 × 10^−10^	3.31 × 10^−8^
hsa-miR-27b-3p	98.85	6.61 × 10^−10^	3.34 × 10^−8^
hsa-miR-3196	62.73	7.00 × 10^−10^	3.53 × 10^−8^
hsa-miR-197-5p	19.83	8.26 × 10^−10^	4.11 × 10^−8^

**Table 4:** Expression profile analysis of positive miRNAs complete scaffold versus control (undifferentiated ASC).

Transcript ID (Array Design)	Fold Change	P-Val	FDR P-Val
hsa-miR-222-3p	−8.88	7.79 × 10^−10^	2.82 × 10^−6^
hsa-miR-145-5p	4.69	3.17 × 10^−8^	5.23 × 10^−5^
hsa-miR-221-3p	−6.59	6.84 × 10^−8^	6.04 × 10^−5^
hsa-miR-3135b	5.28	7.26 × 10^−8^	6.26 × 10^−5^
hsa-miR-23b-5p	−15.69	1.87 × 10^−7^	0.0001
hsa-miR-4721	−10.22	2.05 × 10^−7^	0.0002
hsa-miR-6846-5p	−3.4	4.62 × 10^−7^	0.0003
hsa-miR-184	12.26	8.25 × 10^−7^	0.0004
hsa-miR-214-5p	−5.12	1.07 × 10^−6^	0.0005
hsa-miR-99a-5p	3.58	2.24 × 10^−6^	0.0007
hsa-miR-296-3p	−2.9	3.66 × 10^−6^	0.0011
hsa-miR-146a-5p	−8.91	5.99 × 10^−6^	0.0015
hsa-miR-6741-5p	−4.14	1.70 × 10^−5^	0.004
hsa-miR-1229-5p	−3.38	1.90 × 10^−5^	0.0044
hsa-miR-34a-3p	−3.28	2.09 × 10^−5^	0.0047
hsa-miR-1224-5p	−4.05	2.72 × 10^−5^	0.006
hsa-miR-3663-3p	−7.27	3.37 × 10^−5^	0.0073
hsa-miR-150-3p	−7.05	4.43 × 10^−5^	0.0088
hsa-miR-23a-5p	−4.75	4.89 × 10^−5^	0.0094
hsa-miR-6860	−2.81	5.98 × 10^−5^	0.0096
hsa-miR-3157-3p	2.41	6.66 × 10^−5^	0.0097
hsa-miR-4685-5p	−3.23	7.81 × 10^−5^	0.0109
hsa-miR-615-5p	−2.51	9.49 × 10^−5^	0.0128
hsa-miR-1275	−2.83	0.0001	0.0139
hsa-miR-1587	−3.15	0.0001	0.0157
hsa-miR-874-3p	−5.11	0.0001	0.0157
hsa-miR-6742-5p	−2.62	0.0001	0.0157
hsa-miR-708-5p	−8.08	0.0001	0.0157
hsa-miR-3679-5p	−3.83	0.0002	0.017
hsa-miR-574-3p	2.07	0.0002	0.0175
hsa-miR-1182	−2.12	0.0002	0.0183
hsa-miR-195-5p	11.16	0.0002	0.0183
hsa-miR-6165	−3.16	0.0002	0.0219
hsa-miR-143-3p	2.67	0.0002	0.0221
hsa-miR-7107-5p	2.12	0.0003	0.0222
hsa-miR-30b-5p	−8.6	0.0003	0.0222
hsa-miR-181a-5p	−2.27	0.0003	0.0236
hsa-miR-328-3p	−3.34	0.0003	0.0246
hsa-miR-4269	−2.49	0.0004	0.0278
hsa-miR-6889-5p	−2.32	0.0004	0.0288
hsa-miR-501-3p	3.37	0.0004	0.0297
hsa-miR-3180-3p	−2.35	0.0004	0.0297
hsa-miR-1909-3p	−2.52	0.0004	0.0308
hsa-miR-6824-5p	−3.06	0.0004	0.0312
hsa-miR-502-3p	3.87	0.0005	0.0315
hsa-miR-557	−3.28	0.0005	0.0321
hsa-miR-6870-5p	−4.78	0.0005	0.0344
hsa-miR-933	−4.31	0.0005	0.035
hsa-miR-197-5p	−2.24	0.0006	0.0401
hsa-miR-500a-3p	3.69	0.0006	0.0418
hsa-miR-421	−4.08	0.0007	0.0434
hsa-miR-504-3p	−2.8	0.0008	0.0481

## Data Availability

Original data will be made available upon request.

## Supplementary Materials

The following supporting information can be downloaded at: https://www.mdpi.com/article/10.3390/ijms25126762/s1. References [65,66] are cited in the Supplementary Materials.

## References

[1] Guyton A.C., Hall J.E. (2017). Tratado de Fisiologia Médica.

[2] Norgren L., Hiatt W.R., Dormandy J.A., Nehler M.R., Harris K.A., Fowkes F.G., TASC II Working Group (2007). Inter-Society Consensus for the Management of Peripheral Arterial Disease (TASC II). Eur. J. Vasc. Endovasc. Surg..

[3] Jaff M.R., White C.J., Hiatt W.R., Fowkes G.R., Dormandy J., Razavi M., Reekers J., Norgren L., TASC Steering Committee (2015). An Update on Methods for Revascularization and Expansion of the TASC Lesion Classification to Include Below-the-Knee Arteries: A Supplement to the Inter-Society Consensus for the Management of Peripheral Arterial Disease (TASC II). Vasc. Med..

[4] Allison M.A., Ho E., Denenberg J.O., Langer R.D., Newman A.B., Fabsitz R.R., Criqui M.H. (2007). Ethnic-specific prevalence of peripheral arterial disease in the United States. Am. J. Prev. Med..

[5] Selvin E., Erlinger T.P. (2004). Prevalence of and risk factors for peripheral arterial disease in the United States: Results from the National Health and Nutrition Examination Survey, 1999–2000. Circulation.

[6] Criqui M.H., Aboyans V. (2015). Epidemiology of peripheral artery disease. Circ. Res..

[7] Deb S., Wijeysundera H.C., Ko D.T., Tsubota H., Hill S., Fremes S.E. (2013). Coronary artery bypass graft surgery vs percutaneous interventions in coronary revascularization: A systematic review. JAMA.

[8] Rocco K.A., Maxfield M.W., Best C.A., Dean E.W., Breuer C.K. (2014). In vivo applications of electrospun tissue-engineered vascular grafts: A review. Tissue Eng. Part B Rev..

[9] Sukovatykh B., Belikov L., Sukovatykh M., Sidorov D., Inarkhov M., Inokhodova E. (2016). Results of using various methods of autovenous femoropopliteal bypass grafting below the knee-joint fissure. Angiol. Vasc. Surg..

[10] Gaudino M., Taggart D., Suma H., Puskas J.D., Crea F., Massetti M. (2015). The choice of conduits in coronary artery bypass surgery. J. Am. Coll. Cardiol..

[11] Cleary M.A., Geiger E., Grady C., Best C., Naito Y., Breuer C. (2012). Engenharia de tecidos vasculares: A próxima geração. Trends Mol. Med..

[12] Zampetaki A., Mayr M. (2012). MicroRNAs in vascular and metabolic disease. Circ. Res..

[13] Leroy O., Meybeck A., Sarraz-Bournet B., d’Elia P., Legout L. (2012). Vascular graft infections. Curr. Opin. Infect. Dis..

[14] Newby A.C., Zaltsman A.B. (1999). Fibrous cap formation or destruction-the critical importance of vascular smooth muscle cell proliferation, migration and matrix formation. Cardiovasc. Res..

[15] Marx S.O., Totary-Jain H., Marks A.R. (2011). Vascular smoothmuscle cell proliferation in restenosis. Circ. Cardiovasc. Interv..

[16] Doran A.C., Meller N., McNamara C.A. (2008). Role of smoothmuscle cells in the initiation and early progression of atherosclerosis. Arterioscler. Thromb. Vasc. Biol..

[17] Owens G.K., Kumar M.S., Wamhoff B.R. (2004). Molecular regulation of vascular smooth muscle cell differentiation in development and disease. Physiol. Rev..

[18] Andrae J., Gallini R., Betsholtz C. (2008). Role of platelet-derived growth factors in physiology and medicine. Genes Dev..

[19] Tiwari A., Mukherjee B., Dixit M. (2018). MicroRNA Key to Angiogenesis Regulation: MiRNA Biology and Therapy. Curr. Cancer Drug Targets.

[20] Carmeliet P., Jain R.K. (2011). Molecular mechanisms and clinical applications of angiogenesis. Nature.

[21] Chung A.S., Lee J., Ferrara N. (2010). Targeting the tumour vasculature: Insights from physiological angiogenesis. Nat. Rev. Cancer.

[22] Ribatti D., Conconi M.T., Nussdorfer G.G. (2007). Nonclassic endogenous novel regulators of angiogenesis. Pharmacol. Rev..

[23] Kuehbacher A., Urbich C., Zeiher A.M., Dimmeler S. (2007). Role of dicer and drosha for endothelial microRNA expression and angiogenesis. Circ. Res..

[24] Wang S., Olson E.N. (2009). AngiomiRs-Key regulators of angiogenesis. Curr. Opin. Genet. Dev..

[25] Yoo J.K., Kim J., Choi S.J., Noh H.M., Kwon Y.D., Yoo H., Yi H.S., Chung H.M., Kim J.K. (2012). Discovery and characterization of novel microRNAs during endothelial differentiation of human embryonic stem cells. Stem Cells Dev..

[26] Tili E., Michaille J.J., Calin G.A. (2008). Expressão e função de micro-RNAs em células imunes durante o estado normal ou de doença. Int. J. Med. Sci..

[27] Song Z., Li G. (2010). Role of specific microRNAs in regulation of vascular smooth muscle cell differentiation and the response to injury. J. Cardiovasc. Transl. Res..

[28] Zhang C. (2010). MicroRNAs in vascular biology and vascular disease. J. Cardiovasc. Transl. Res..

[29] Gallach S., Calabuig-Fariñas S., Jantus-Lewintre E., Camps C. (2014). MicroRNAs: Promising new antiangiogenic targets in cancer. Biomed. Res. Int..

[30] Chang S.-H., Hla T. (2011). Gene regulation by RNA binding proteins and microRNAs inangiogenesis. Trends Mol. Med..

[31] Carmeliet P., Jain R.K. (2000). Angiogenesis in cancer and other diseases. Nature.

[32] Folkman J. (1989). Induction of angiogenesis during the transition from hyperplasia toneoplasia. Nature.

[33] Folkman J. (1971). Tumor angiogenesis: Therapeutic implications. N. Engl. J. Med..

[34] DeCicco-Skinner K.L., Henry G.H., Cataisson C., Tabib T., Gwilliam J.C., Watson N.J., Bullwinkle E.M., Falkenburg L., O’Neill R.C., Morin A. (2014). Endothelial cell tube formation assay for the in vitro study of angiogenesis. J. Vis. Exp..

[35] Beavers K.R., Nelson C.E., Duvall C.L. (2015). MiRNA inhibition in tissue engineering and regenerative medicine. Adv. Drug Deliv. Rev..

[36] Chung A.S., Ferrara N. (2011). Developmental and pathological angiogenesis. Annu. Rev. Cell Dev. Biol..

[37] Dudley A.C., Griffioen A.W. (2023). Pathological angiogenesis: Mechanisms and therapeutic strategies. Angiogenesis.

[38] Chen E.Y., Tan C.M., Kou Y., Duan Q., Wang Z., Meirelles G.V., Clark N.R., Ma’ayan A. (2013). Enrichr: Ferramenta de análise de enriquecimento de lista de genes HTML5 interativa e colaborativa. BMC Bioinform..

[39] Kuleshov M.V., Jones M.R., Rouillard A.D., Fernandez N.F., Duan Q., Wang Z., Koplev S., Jenkins S.L., Jagodnik K.M., Lachmann A. (2016). Enrichr: Um conjunto abrangente de genes atualização do servidor web de análise de enriquecimento 2016. Nucleic Acids Res..

[40] Xie Z., Bailey A., Kuleshov M.V., Clarke D.J.B., Evangelista J.E., Jenkins S.L., Lachmann A., Wojciechowicz M.L., Kropiwnicki E., Jagodnik K.M. (2021). Gene definiu descoberta de conhecimento com Enrichr. Protoc. Atuais.

[41] Kang T., Jones T.M., Naddell C., Bacanamwo M., Calvert J.W., Thompson W.E., Bond V.C., Chen Y.E., Liu D. (2016). Adipose-Derived Stem Cells Induce Angiogenesis via Microvesicle Transport of miRNA-31. Stem Cells Transl. Med..

[42] Gilbert T.W., Sellaro T.L., Badylak S.F. (2006). Decellularization of tissues and organs. Biomaterials.

[43] Wang Y., Blelloch R. (2009). Cell cycle regulation by MicroRNAs in embryonic stem cells. Cancer Res..

[44] Bertanha M., Sobreira M.L., Bovolato A.L.C., Rinaldi J.C., Reis P.P., Moroz A., Moraes L.N., Deffune E. (2017). Ultrastructural analysis and residual DNA evaluation of rabbit vein scaffold. Acta Cir. Bras..

[45] Bertanha M., Moroz A., Jaldin R.G., Silva R.A., Rinaldi J.C., Golim M.A., Felisbino S.L., Domingues M.A., Sobreira M.L., Reis P.P. (2014). Morphofunctional characterization of decellularized vena cava as tissue engineering scaffolds. Exp. Cell Res..

[46] Rodrigues L.D.S., Bovolato A.L.C., Silva B.E., Chizzolini L.V., Cruz B.L.D., Moraes M.P.T., Lourenção P.L.T.A., Bertanha M. (2021). Quantification of adhesion of mesenchymal stem cells spread on decellularized vein scaffold. Acta Cir. Bras..

[47] Secondo M.T.S., Rodrigues L.D.S., Ramos L.P.M., Bovolato A.L.C., Rodriguez-Sanchez D.N., Sobreira M.L., Moraes M.P.T., Bertanha M. (2022). Evaluation of Biointegration and Inflammatory Response to Blood Vessels Produced by Tissue Engineering—Experimental Model in Rabbits. Biomolecules.

[48] Bertanha M., Moroz A., Almeida R., Alves F.C., Valério M.J.A., Moura R., Domingues M.A.C., Sobreira M.L., Deffune E. (2014). Tissue-engineered blood vessel substitute by reconstruction of endothelium using mesenchymal stem cells induced by platelet growth factors. J. Vasc. Surg..

[49] Garcia L.R., Garzesi A.M., Brito F.D.S., Felicio M.L., Bertanha M. (2023). Scaffolds for Use in Blood Vessel Bioengineering: What are the Prospects?. Arq. Bras. Cardiol..

[50] Wang Y., Blelloch R. (2011). Cell cycle regulation by microRNAs in stem cells. Results Probl. Cell Differ..

[51] Dalton S. (2009). Expondo dimensões ocultas do controle do ciclo de células-tronco embrionárias. Cell Stem Cell.

[52] Hao J., Duan F.F., Wang Y. (2017). MicroRNAs and RNA binding protein regulators of microRNAs in the control of pluripotency and reprogramming. Curr. Opin. Genet. Dev..

[53] Li N., Long B., Han W., Yuan S., Wang K. (2017). microRNAs: Important regulators of stem cells. Stem Cell Res. Ther..

[54] Lichner Z., Páll E., Kerekes A., Pállinger É., Maraghechi P., Bősze Z., Gócza E. (2011). The miR-290-295 cluster promotes pluripotency maintenance by regulating cell cycle phase distribution in mouse embryonic stem cells. Differentiation.

[55] Houbaviy H.B., Murray M.F., Sharp P.A. (2003). Embryonic stem cell-specific microRNAs. Dev. Cell.

[56] Luo Z., Wen G., Wang G., Pu X., Ye S., Xu Q., Wang W., Xiao Q. (2013). MicroRNA-200C and -150 play an important role in endothelial cell differentiation and vasculogenesis by targeting transcription repressor ZEB1. Stem Cells.

[57] Yang W.J., Yang D.D., Na S., Sandusky G.E., Zhang Q., Zhao G. (2005). Dicer is required for embryonic angiogenesis during mouse development. J. Biol. Chem..

[58] Zhao Z., Sun W., Guo Z., Zhang J., Yu H., Liu B. (2020). Mechanisms of lncRNA/microRNA interactions in angiogenesis. Life Sci..

[59] Fish J.E., Santoro M.M., Morton S.U., Yu S., Yeh R.F., Wythe J.D., Ivey K.N., Bruneau B.G., Stainier D.Y., Srivastava D. (2008). miR-126 regulates angiogenic signaling and vascular integrity. Dev. Cell.

[60] Wang S., Aurora A.B., Johnson B.A., Qi X., McAnally J., Hill J.A., Richardson J.A., Bassel-Duby R., Olson E.N. (2008). The endothelial-specific microRNA miR-126 governs vascular integrity and angiogenesis. Dev. Cell.

[61] Chen Y., Gorski D.H. (2008). Regulation of angiogenesis through a microRNA (miR-130a) that down-regulates antiangiogenic homeobox genes GAX and HOXA5. Blood.

[62] Fasanaro P., D’Alessandra Y., Di Stefano V., Melchionna R., Romani S., Pompilio G., Capogrossi M.C., Martelli F. (2008). MicroRNA-210 modulates endothelial cell response to hypoxia and inhibits the receptor tyrosine kinase ligand Ephrin-A3. J. Biol. Chem..

[63] Chomczynski P., Sacchi N. (1987). Single-step method of RNA isolation by acid guanidinium thiocyanate-phenol-chloroform extraction. Anal. Biochem..

[64] Chomczynski P., Sacchi N. (2006). The single-step method of RNA isolation by acid guanidinium thiocyanate-phenol-chloroform extraction: Twenty-something years on. Nat. Protoc..

[65] Docheva D., Padula D., Popov C., Mutschler W., Clausen-Schaumann H., Schieker M. (2008). Researching into the cellular shape, volume and elasticity of mesenchymal stem cells, osteoblasts and osteosarcoma cells by atomic force microscopy. J. Cell. Mol. Med..

[66] Gugjoo M.B., Kinjavdekar A.P., Aithal H.P., Ansari M.M., Pawde A.M., Sharma G.T. (2015). Isolation, Culture, and Characterization of New Zealand White Rabbit Mesenchymal Stem Cells Derived from Bone Marrow. Asian J. Anim. Vet. Adv..

