# Supplementary material for: Deciphering Key microRNA Regulated Pathways in Tissue-Engineered Blood Vessels: Implications for Vascular Scaffold Production
Source: Int J Mol Sci. 2024 Jun 20;25(12):6762. doi: 10.3390/ijms25126762 (PMC11203763; doi:10.3390/ijms25126762)
Supplement: Supplementary file 1 [file ijms-25-06762-s001.zip › ijms-3024412-supplementary.pdf]

Supplemental Files

Index

Supplemental File S1. Obtaining adipose tissue and expanding ASCs

Supplemental File S2. Vein decellularization protocol

Supplemental File S3. Endothelial differentiation of ASCs in the scaffold

Supplemental File S4. Differentiation into smooth muscle cells

Supplemental Table 1. Network of molecular pathways of the endothelialized scaffold versus control

Supplemental Table 2.Network of molecular pathways of the scaffold endothelialized with smooth muscle versus control.

SupplementaL Table 3. Molecular pathway network of endothelial cells versus undifferentiated MSC

Supplemental Table 4. Molecular pathway network of smooth muscle cells versus undifferentiated MSCs.

Supplemental file S1. Obtaining adipose tissue and expanding ASCs

In order to obtain ASCs, the animals were anesthetized, then 2 g of AT was surgically removed from the interscapular region and stored in a conical tube containing a solution of N-2-hydroxyethylpiperazine-N-2-ethanesulfonic acid (HEPES) supplemented with penicillin, 100 mg/mL streptomycin, and 25 mg/mL amphotericin B (2 mmol/L 1-glutamine; Invitrogen™, Waltham, MA, USA). ASCs were obtained by enzymatic dissociation with collagenase type I (Invitrogen™, Waltham, MA, USA). Cell culture procedures were performed with an initial cell count of 6 × 104 cells/cm2, obtained from 12 fragments of adipose tissue. These cells were seeded and expanded in 25 cm2 culture flasks using Dulbecco's modified Eagle's medium (DMEM), supplemented with 10% fetal bovine serum (FBS), 100 U/mL penicillin, 100 mg/mL streptomycin, 25 mg/mL amphotericin B (2 mmol/L of l-glutamine; Invitrogen™, Waltham, MA, USA), 1% (v/v) of minimal essential medium (MEM), an essential amino acid solution (Invitrogen™, Waltham, MA, USA), and 0.5% (v/v) of 10 mM MEM nonessential amino acid solution (Invitrogen™, Waltham, MA, USA) until the number of cells required for the entire study was reached. ASCs were analyzed phenotypically by flow cytometry (FC) using CD45, CD44, CD90, and CD11b, and by tri-lineage differentiation techniques (StemPro™ adipogenesis, chondrogenesis, and osteogenesis differentiation kits; Invitrogen, Waltham, MA, USA).

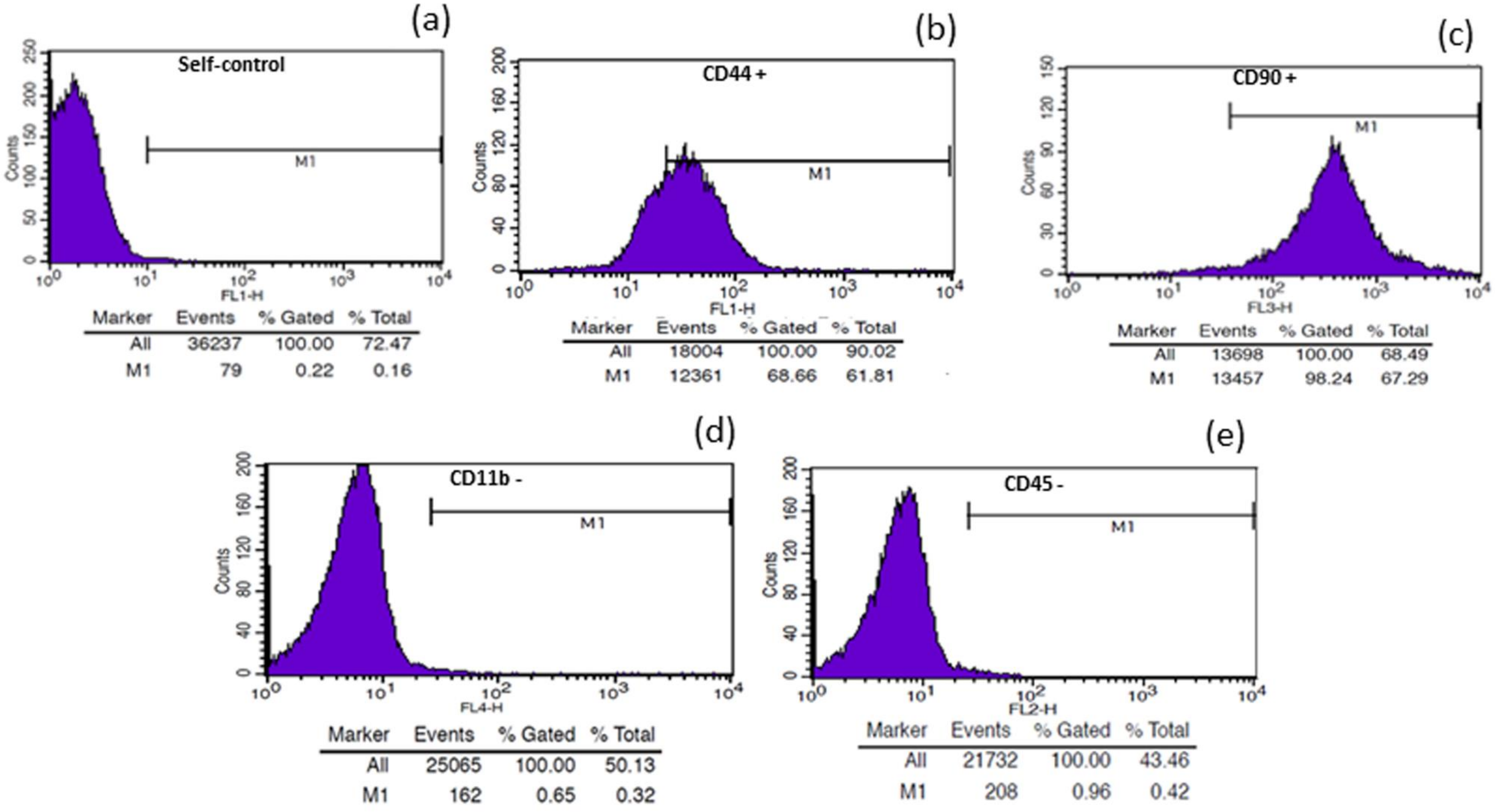

Supplemental Figure S1.1 Representative phenotypic characterization by flow cytometry for cell surface markers from one rabbit ASC. Histogram **(a)** shows self-control for the evaluation of cell autofluorescence. Histograms (**b)** and **(c)** show MSC markers CD44 and CD90, respectively, with positive peaks. Histograms **(d)** and **(e)** show hemopoietic and immune markers CD45 and CD11b, respectively, with no peaks (negative). All markers used correspond to those described in the literature.

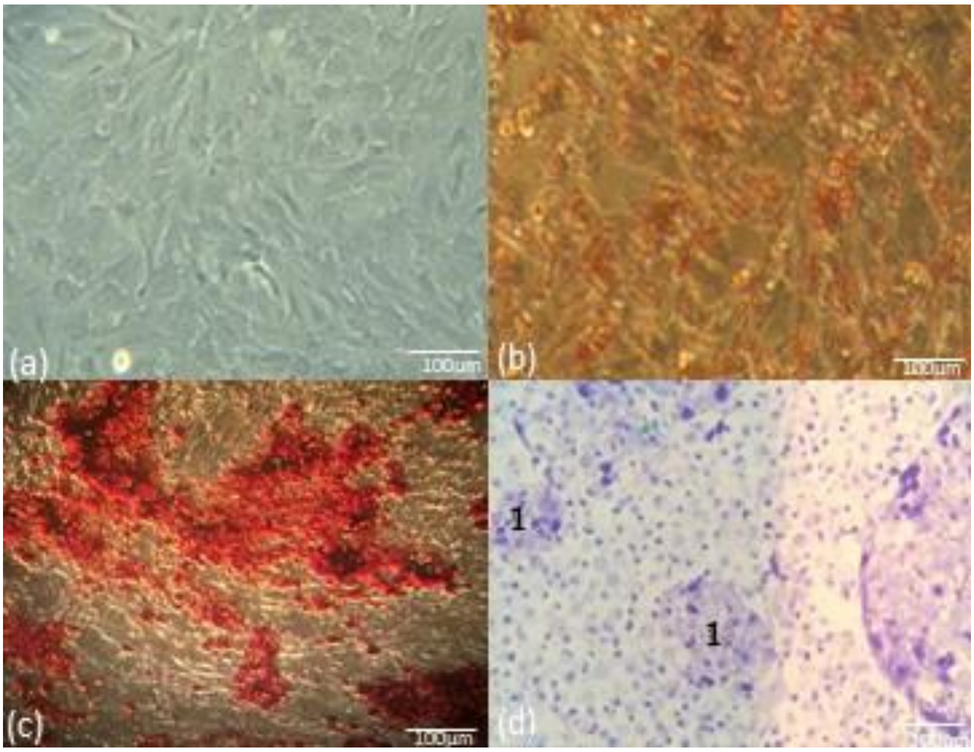

Supplemental Figure S1.2 Tri-lineage Differentiation. **(a)**. Undifferentiated mesenchymal stem cells; **(b)**. Adipocyte differentiation: The appearance of adipocytes after staining with Oil Red®, where lipid droplets with reddish content can be observed; **(c)**.Bone differentiation: the differentiated bone tissue in the presence of an inducing medium, were stained with Alizarin Red®. Red areas indicate a large confluence of calcium deposits, evidence of bone trabeculae. **(d)**. Cartilage differentiation: Histological section of chondrogenic differentiation from ACS stained with Alcian Blue; 1 – isogenic core.

[65] Docheva D, Padula D, Popov C, Mutschler W, Clausen-Schaumann H, Schieker M. Researching into the cellular shape, volume and elasticity of mesenchymal stem cells, osteoblasts and osteosarcoma cells by atomic force microscopy. J. Cell Mol. Med. 2008;12: 537-552.
[66] Gugjoo MB, Kinjavdekar AP, Aithal HP, Ansari MM, Pawde AM, Sharma GT. Isolation, Culture, and Characterization of New Zealand White Rabbit Mesenchymal Stem Cells Derived from Bone Marrow. X Asian Journal of Animal and Veterinary Advances. 2015;10: 537-548.

Suplementary file S2. Vein decellularization protocol

Material

- Conical tube
- Injection water
- Sodium duodecyl sulfate (SDS) 1%
- Clean and fragmented sample

Solution preparation Sodium duodecyl sulfate (SDS) 1%: 200mL of ultrafiltered H20 + 2.5g SDS (molecular weight 288.38, concentration 0.35 M, Sigma Aldrich, San Luis, Missouri, USA)

Ps. Each conical tube must be filled with 25 mL of the 1% SDS solution.

Procedure

In a laminar flow chamber, sterile environment, the samples (veins) must go through the cleaning procedure to ensure that there are no remains of blood and fat compound, then separated into fragments according to the study protocol. The fragmented samples will be stored at -80ºC until the moment of decellularization.

Before the decellularization process, the fragments will be thawed at room temperature, washed with saline solution, and stored 5 samples per conical tube containing 25mL of 1% SDS solution, seal the lid with parafilm to prevent leakage. Place the tubes in a horizontal position on the shaker tray and fix them with tape, keep stirring at 1200rpm for 2 hours at 37ºC (Shaker News Brunswick Scientific® with the controlled temperature at 37ºC). The fragments went through a cycle of 3 washes with sterile saline solution and were conditioned and preserved in a refrigerator at 4ºC in a sterile solution containing antibiotic and antifungal until the moment of use in culture.

Observations: Use mask for handling SDS, mucosa irritant substance.

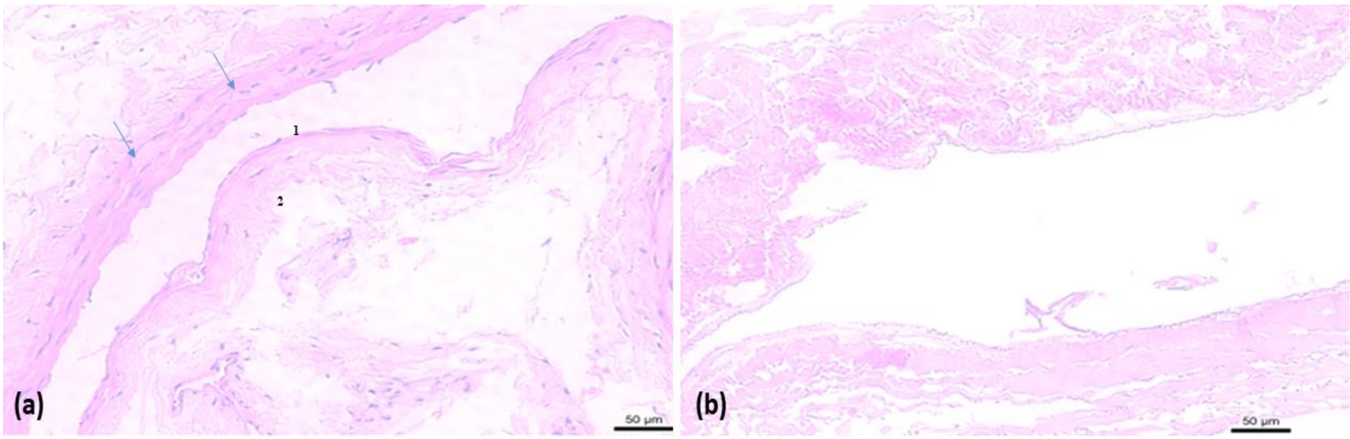

Supplemental Figure S2. Evidence of inferior vena cava decellularization. H&E staining of **(a)** Vein in natura; **(b)** Successful decellularization of the inferior vena cava.

[44] Bertanha M, Sobreira ML, Bovolato ALC, Rinaldi JC, Reis PP, Moroz A, Moraes LN, Deffune E. Ultrastructural analysis and residual DNA evaluation of rabbit vein scaffold. Acta Cir Bras. 2017 Sep;32(9):706-711.

[45] Bertanha M, Moroz A, Jaldin RG, Silva RA, Rinaldi JC, Golim MA, Felisbino SL, Domingues MA, Sobreira ML, Reis PP, Deffune E. Morphofunctional characterization of decellularized vena cava as tissue engineering scaffolds. Exp Cell Res. 2014 Aug 1;326(1):103-11.

[46] Rodrigues LDS, Bovolato ALC, Silva BE, Chizzolini LV, Cruz BLD, Moraes MPT, Lourenção PLTA, Bertanha M. Quantification of adhesion of mesenchymal stem cells spread on decellularized vein scaffold. Acta Cir Bras. 2021 Nov 5;36(10):e361001.

Supplemental file S3. Endothelial differentiation of ASCs in the scaffold

DMEM Culture Medium

• 5% SFB

• 10ng/mL VEGF (Vascular Endothelial Growth Factor)

• 50ng/mL bFGF (Basic Fibroblast Growth Factor)

• 20ng/mL IGF (Insulin Growth Factor)

• 20ng/mL EGF (Epidermal Growth Factor)

• 24-well plate (2mL per well), 48mL per plate is used (4 weeks of cultivation uses 384mL of differentiation culture medium)

Prepare the culture medium as planned to be used in volume in the protocol. DMEN culture medium supplemented with 5% SFB, 10ng/mL VEGF, 50ng/mL bFGF, 20ng/mL IGF, 20ng/Ml EGF. Each scaffold received an aliquot of 20 uL of undifferentiated ASCs that were applied to the lumen using a micropipette. The scaffolds were placed in a 24-well plate with cell differentiation culture medium. The culture plates were maintained in a 5% CO2 incubator, in a controlled manner, at 37oC, with medium changes every two days. To confirm the endothelial differentiation of ASCs into endothelium within the scaffold, slides were prepared for immunohistochemistry: anti-CD146.

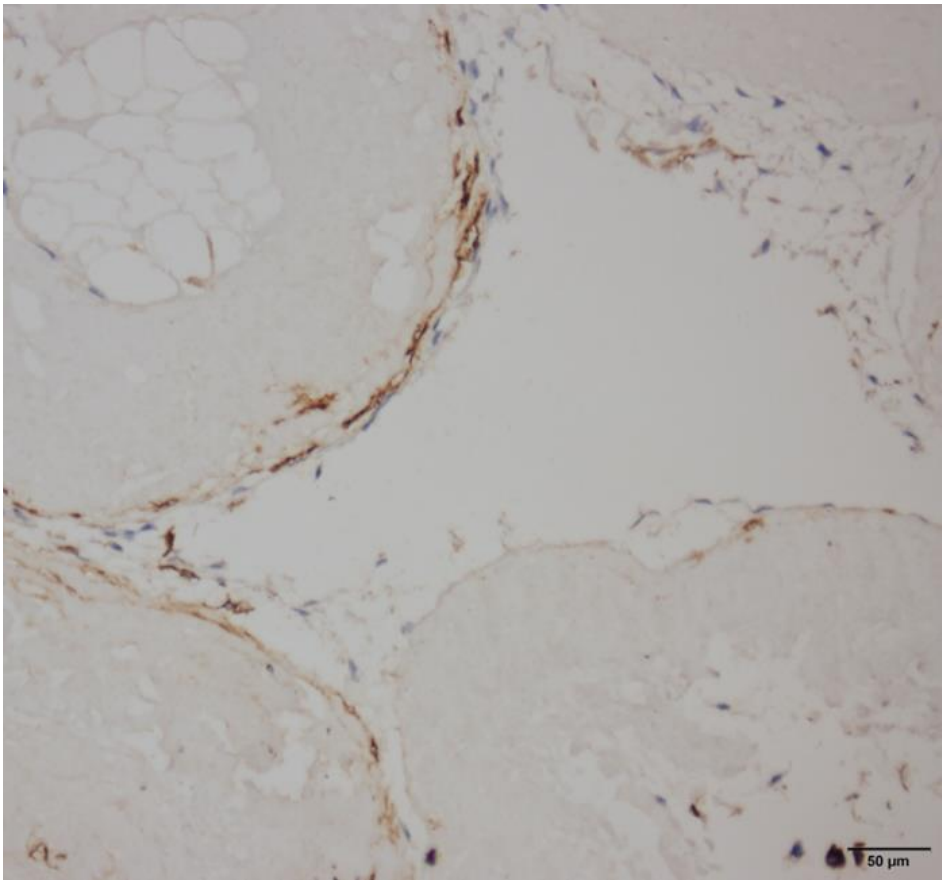

Supplemental Figure S3. Confirmation of endothelial recellularization in the scaffold by immunohistochemistry

SupplementaL file S4. Differentiation into smooth muscle cells

To differentiate ASCs into smooth muscle, we will use previously expanded cultures of undifferentiated ASC with 20% confluency in 175cm2 bottles to initiate cell differentiation, which were treated with M199 culture medium supplemented with 5% SFB, 2.5 ng/ mL of human recombinant BMP4 and 5 ng/mL of TGF-1. This culture was maintained for 3 weeks, kept in a 5% CO2 incubator, in a controlled manner, at 37oC, with medium changes every two days. The muscle cells produced were detached from the culture flask with trypsin for use in the seeding stage on the endothelialized scaffold and a sample was used for immunohistochemical analysis, creating a cell "pellet". To confirm the differentiation of ASCs into smooth muscle cells, slides were prepared for immunohistochemistry: anti-α actin 1a4.

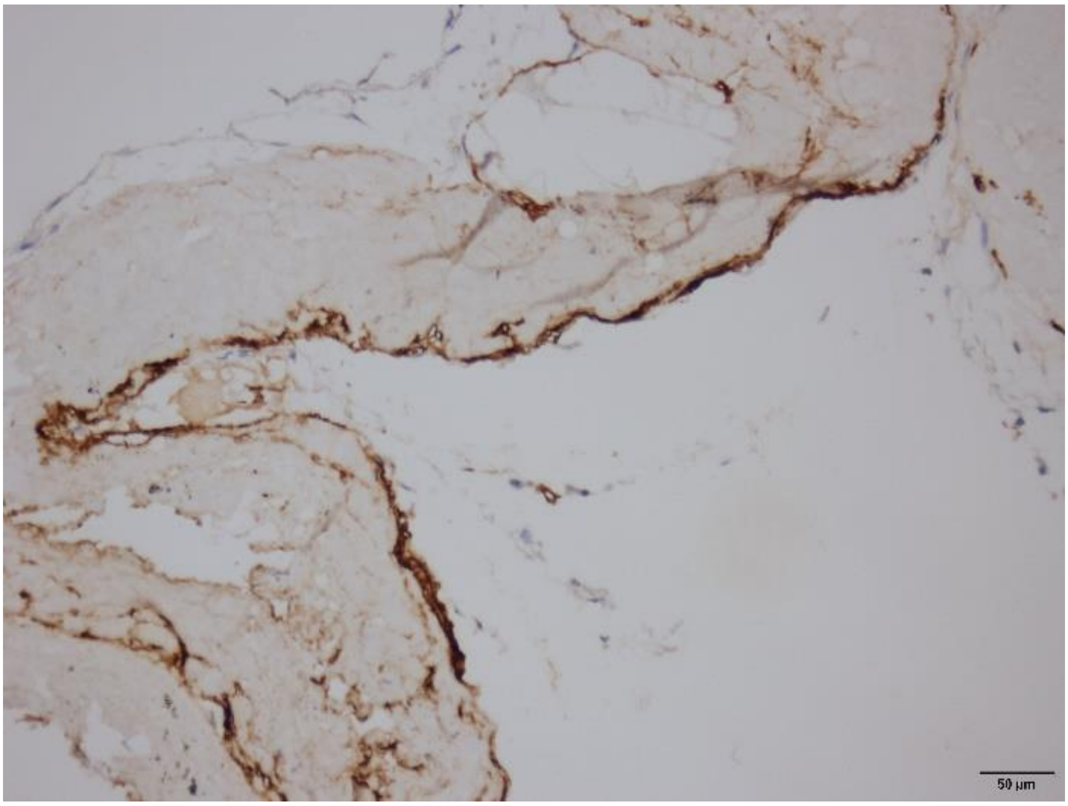

Supplementary Figure S4. Confirmation of smooth muscle recellularization in the scaffold by immunohistochemistry

Supplemental Table S1. Network of molecular pathways of the endothelialized scaffold versus control

Supplemental Tabela 1. Network of molecular pathways of the endothelialized scaffold versus control.

Term	P-value	Genes	miRNA
Vascular system	0.010736	VEZT	hsa-let-7a-5p
Vascular system	0.010736	BMPR2	hsa-let-7a-5p; hsa-miR-143-3p
Vascular system	0.010736	SNAP23	hsa-miR-143-3p; hsa-let-7a-5p
Vascular system	0.010736	BZW1	hsa-let-7a-5p; hsa-let-7c-5p; hsa-let-7i-5p
Vascular system	0.010736	CCND1	hsa-miR-6732-5p;
Vascular system	0.010736	LAMP2	hsa-miR-145-5p; hsa-miR-8075
Vascular system	0.010736	TNPO1	hsa-miR-27b-3p; hsa-miR-26a-5p; hsa-let-7a-5p
Vascular system	0.010736	RAB8B	hsa-miR-143-3p; hsa-miR-6808-3p
Vascular system	0.010736	ACTR2	hsa-miR-6808-3p
Vascular system	0.010736	EDN1	hsa-let-7i-5p; hsa-let-7a-5p; hsa-let-7c-5p; hsa-miR-143-3p; hsa-miR-191-5p
Vascular system	0.010736	DUSP6	hsa-miR-145-5p;
Vascular system	0.010736	FOXP1	hsa-miR-191-5p; hsa-miR-6808-3p
Vascular system	0.010736	DKK3	hsa-miR-6732-5p;
Vascular system	0.010736	ENAH	hsa-miR-191-5p; hsa-miR-6808-3p
Vascular system	0.010736	RCN1	hsa-miR-145-5p;
Vascular system	0.010736	TMEM135	hsa-miR-145-5p; hsa-miR-143-3p
Vascular system	0.010736	SUB1	hsa-miR-455-3p
Vascular system	0.010736	MAP1B	hsa-miR-143-3p
Vascular system	0.010736	PAPPA	hsa-let-7a-5p; hsa-let-7c-5p; hsa-let-7i-5p
Vascular system	0.010736	ADAM9	hsa-miR-191-5p; hsa-miR-26a-5p
Vascular system	0.010736	BCAT1	hsa-let-7a-5p; hsa-let-7c-5p; hsa-let-7i-5p
Vascular system	0.010736	TP53	hsa-let-7a-5p; hsa-let-7c-5p; hsa-miR-6732-5p; hsa-miR-27b-3p; hsa-miR-455-3p; hsa-miR-143-3p; hsa-miR-8075; hsa-miR-6808-3p; hsa-miR-145-5p; hsa-let-7i-5p
Vascular system	0.010736	LRRC59	hsa-miR-143-3p
Vascular system	0.010736	SRSF1	hsa-let-7a-5p; hsa-miR-26a-5p; hsa-miR-27b-3p
Vascular system	0.010736	ADD3	hsa-miR-143-3p; hsa-miR-145-5p
Vascular system	0.010736	FAM214B	hsa-let-7a-5p; hsa-let-7c-5p
Vascular system	0.010736	THBS1	hsa-let-7a-5p; hsa-let-7i-5p; hsa-let-7c-5p; hsa-miR-6732-5p; hsa-miR-455-3p
Vascular system	0.010736	G3BP1	hsa-miR-27b-3p; hsa-miR-455-3p; hsa-let-7a-5p;
Vascular system	0.010736	CD59	hsa-miR-5000-5p;

Vascular system	0.010736	SLC38A2	hsa-miR-145-5p; hsa-miR-26a-5p; hsa-miR-145-5p; hsa-miR-143-3p; hsa-miR-5000-5p
Vascular system	0.010736	SPTBN1	hsa-miR-6732-5p; hsa-let-7a-5p; hsa-miR-145-5p; hsa-miR-6732-5p;
Vascular system	0.010736	RRM2	hsa-let-7a-5p; hsa-let-7i-5p;
Vascular system	0.010736	HMGA1	hsa-let-7c-5p; hsa-let-7i-5p; hsa-miR-26a-5p
Vascular system	0.010736	COL1A1	hsa-let-7i-5p; hsa-miR-143-3p; hsa-miR-6732-5p;
Vascular system	0.010736	UFM1	hsa-miR-6808-3p
Vascular system	0.010736	DLC1	hsa-let-7c-5p; hsa-let-7a-5p
Vascular system	0.010736	COL5A2	hsa-miR-143-3p; hsa-let-7i-5p
Vascular system	0.010736	CALU	hsa-miR-143-3p
Vascular system	0.010736	CALM1	hsa-miR-6808-3p; hsa-miR-191-5p; hsa-miR-143-3p
Vascular system	0.010736	EIF4G2	hsa-let-7a-5p; hsa-let-7c-5p; hsa-let-7i-5p; hsa-miR-26a-5p
Vasculature	0.022924	EDN1	hsa-let-7i-5p; hsa-let-7a-5p; hsa-let-7c-5p; hsa-miR-143-3p; hsa-miR-191-5p
Vasculature	0.022924	BMPR2	hsa-let-7a-5p; hsa-miR-143-3p
Vasculature	0.022924	PLXND1	hsa-let-7i-5p;
Vasculature	0.022924	FZD4	hsa-let-7c-5p; hsa-miR-6732-5p
Vasculature	0.022924	PDGFB	hsa-let-7i-5p; hsa-miR-6808-3p; hsa-let-7a-5p; hsa-miR-27b-3p; hsa-miR-455-3p; hsa-miR-143-3p; hsa-miR-145-5p
Vasculature	0.022924	PDE5A	hsa-miR-5000-5p; hsa-let-7a-5p; hsa-miR-191-5p
Vasculature	0.022924	IGF1	hsa-miR-6732-5p; hsa-miR-27b-3p; hsa-miR-455-3p; hsa-miR-145-5p; hsa-let-7i-5p; hsa-let-7a-5p
Vasculature	0.022924	TP53	hsa-let-7a-5p; hsa-let-7c-5p; hsa-miR-6732-5p; hsa-miR-27b-3p; hsa-miR-455-3p; hsa-miR-143-3p; hsa-miR-8075; hsa-miR-6808-3p; hsa-miR-145-5p; hsa-let-7i-5p
Vasculature	0.022924	THBS1	hsa-let-7a-5p; hsa-let-7i-5p; hsa-let-7c-5p; hsa-miR-6732-5p; hsa-miR-455-3p
Vasculature	0.022924	LYVE1	hsa-miR-143-3p
vascular endothelial growth factor receptor signaling pathway (GO:0048010)	0.03301	VAV3	hsa-let-7a-5p; hsa-let-7i-5p; hsa-miR-6808-3p; hsa-miR-27b-3p
vascular endothelial growth factor receptor signaling pathway (GO:0048010)	0.03301	ROCK1	hsa-miR-6808-3p; hsa-miR-455-3p; hsa-let-7a-5p
vascular endothelial growth factor receptor signaling pathway (GO:0048010)	0.03301	ITGB3	hsa-let-7c-5p; hsa-miR-145-5p; hsa-let-7i-5p; hsa-let-7c-5p; hsa-let-7a-5p; hsa-miR-143-3p; hsa-miR-27b-3p
vascular endothelial growth factor receptor signaling pathway (GO:0048010)	0.03301	NCK2	hsa-let-7a-5p; hsa-let-7i-5p; hsa-miR-6808-3p; hsa-miR-145-5p
vascular endothelial growth factor receptor signaling pathway (GO:0048010)	0.03301	ELMO1	hsa-miR-145-5p;
vascular endothelial growth factor receptor signaling pathway (GO:0048010)	0.03301	SULF1	hsa-miR-26a-5p; hsa-miR-143-3p; hsa-let-7i-5p; hsa-miR-27b-3p; hsa-let-7a-5p
vascular endothelial growth factor receptor signaling pathway (GO:0048010)	0.03301	PAK2	hsa-let-7a-5p; hsa-miR-6808-3p; hsa-miR-455-3p; hsa-miR-26a-5p
vascular transport (GO:0010232)	0.038151	INSR	hsa-let-7c-5p; hsa-let-7a-5p; hsa-let-7i-5p; hsa-miR-6732-5p

vascular transport (GO:0010232)	0.038151	SLC1A2	hsa-miR-145-5p; hsa-miR-455-3p; hsa-miR-548a-3p
vascular transport (GO:0010232)	0.038151	SLC6A1	hsa-miR-145-5p; hsa-miR-27b-3p
vascular transport (GO:0010232)	0.038151	SLC38A2	hsa-miR-145-5p; hsa-miR-26a-5p; hsa-miR-145-5p; hsa-miR-143-3p; hsa-miR-5000-5p
venous blood vessel development (GO:0060841)	0.048724	BMPR2	hsa-let-7a-5p; hsa-miR-143-3p
venous blood vessel development (GO:0060841)	0.048724	ACVR2B	hsa-miR-455-3p; hsa-miR-4668-5p
positive regulation of blood vessel endothelial cell migration (GO:0043536)	0.021526	MAP3K3	hsa-miR-6732-5p
positive regulation of blood vessel endothelial cell migration (GO:0043536)	0.021526	SP1	hsa-let-7c-5p; hsa-let-7i-5p; hsa-miR-6732-5p
positive regulation of blood vessel endothelial cell migration (GO:0043536)	0.021526	PDGFB	hsa-let-7i-5p; hsa-miR-6808-3p; hsa-let-7a-5p; hsa-miR-27b-3p; hsa-miR-455-3p; hsa-miR-143-3p; hsa-miR-145-5p
positive regulation of blood vessel endothelial cell migration (GO:0043536)	0.021526	HDAC9	hsa-miR-8075
positive regulation of blood vessel endothelial cell migration (GO:0043536)	0.021526	THBS1	hsa-let-7a-5p; hsa-let-7i-5p; hsa-let-7c-5p; hsa-miR-6732-5p; hsa-miR-455-3p
positive regulation of cell migration (GO:0030335)	0.00144	EDN1;	hsa-let-7i-5p; hsa-let-7a-5p; hsa-let-7c-5p; hsa-miR-143-3p; hsa-miR-191-5p
positive regulation of cell migration (GO:0030335)	0.00144	BMPR2;	hsa-let- 7a-5p;
positive regulation of cell migration (GO:0030335)	0.00144	INSR;	hsa-let-7c-5p; hsa-let-7a-5p; hsa-let-7i-5p; hsa-miR-6732-5p
positive regulation of cell migration (GO:0030335)	0.00144	RDX;	hsa-miR-145-5p ; hsa-let-7a-5p; hsa-let-7c-5p; hsa-let-7i-5p;
positive regulation of cell migration (GO:0030335)	0.00144	SEMA4C;	hsa-let-7a-5p; hsa-let-7i-5p; hsa-let-7c-5p;
positive regulation of cell migration (GO:0030335)	0.00144	PDGFB;	hsa-let-7i-5p; hsa-miR-6808-3p; hsa-let-7a-5p; hsa-miR-27b-3p; hsa-miR-455-3p; hsa-miR-143-3p; hsa-miR-145-5p
positive regulation of cell migration (GO:0030335)	0.00144	SEMA4F	hsa-let-7i-5p
positive regulation of cell migration (GO:0030335)	0.00144	IGF1;	hsa-miR-6732-5p; hsa-miR-27b-3p; hsa-miR-455-3p; hsa-miR-145-5p; hsa-let-7i-5p; hsa-let-7a-5p
positive regulation of cell migration (GO:0030335)	0.00144	ACVR1B;	hsa-miR-455-3p
positive regulation of cell migration (GO:0030335)	0.00144	THBS1;	hsa-let-7a-5p; hsa-let-7i-5p; hsa-let-7c-5p; hsa-miR-6732-5p; hsa-miR-455-3p
positive regulation of cell migration (GO:0030335)	0.00144	TGFBR1;	hsa-let-7a-5p; hsa-let-7c-5p; hsa-let-7i-5p; hsa-miR-6808-3p
positive regulation of cell migration (GO:0030335)	0.00144	CRKL;	hsa-miR-145-5p; hsa-miR-191-5p;
positive regulation of cell migration (GO:0030335)	0.00144	IGF1R;	hsa-let-7a-5p; hsa-let-7c-5p; hsa-let-7i-5p; hsa-miR-143-3p; hsa-miR-145-5p; hsa-miR-6732-5p; hsa-miR-27b-3p
positive regulation of cell migration (GO:0030335)	0.00144	COL1A1;	hsa-let-7i-5p; hsa-miR-143-3p; hsa-miR-6732-5p
positive regulation of cell migration (GO:0030335)	0.00144	PPP3CA;	hsa-miR-145-5p; hsa-miR-143-3p; hsa-let-7i-5p;
positive regulation of cell migration (GO:0030335)	0.00144	PAK1;	hsa-let-7a-5p; hsa-miR-6808-3p;
positive regulation of cell migration (GO:0030335)	0.00144	AKT2;	hsa-let-7i-5p; hsa-miR-6732-5p;
positive regulation of cell migration (GO:0030335)	0.00144	RUFY3;	hsa-miR-6808-3p; hsa-let-7a-5p; hsa-let-7c-5p; hsa-let-7i-5p;
positive regulation of cell migration (GO:0030335)	0.00144	HAS2;	hsa-let-7i-5p; hsa-miR-455-3p
positive regulation of cell migration (GO:0030335)	0.00144	ADAM9;	hsa-miR-191-5p; hsa-miR-26a-5p

positive regulation of cell migration (GO:0030335)	0.00144	HBEGF;	hsa-miR-5000-5p; hsa-let-7a-5p; hsa-miR-6732-5p; hsa-miR-27b-3p; hsa-miR-191-5p
positive regulation of cell migration (GO:0030335)	0.00144	MAP4K4;	hsa-miR-145-5p;
positive regulation of endothelial cell migration (GO:0010595)	0.042942	EDN1;	hsa-let-7i-5p; hsa-let-7a-5p; hsa-let-7c-5p; hsa-miR-143-3p; hsa-miR-191-5p
positive regulation of endothelial cell migration (GO:0010595)	0.042942	BMPR2;	hsa-let-7a-5p; hsa-miR-143-3p
positive regulation of endothelial cell migration (GO:0010595)	0.042942	SP1;	hsa-let-7c-5p; hsa-let-7i-5p; hsa-miR-6732-5p
positive regulation of endothelial cell migration (GO:0010595)	0.042942	ITGB3;	hsa-let-7c-5p; hsa-miR-145-5p; hsa-let-7i-5p; hsa-let-7c-5p; hsa-let-7a-5p; hsa-miR-143-3p; hsa-miR-27b-3p
positive regulation of endothelial cell migration (GO:0010595)	0.042942	PDGFB;	hsa-let-7i-5p; hsa-miR-6808-3p; hsa-let-7a-5p; hsa-miR-27b-3p; hsa-miR-455-3p; hsa-miR-143-3p; hsa-miR-145-5p
positive regulation of endothelial cell migration (GO:0010595)	0.042942	THBS1;	hsa-let-7a-5p; hsa-let-7i-5p; hsa-let-7c-5p; hsa-miR-6732-5p; hsa-miR-455-3p
positive regulation of endothelial cell migration (GO:0010595)	0.042942	FOXP1	hsa-miR-191-5p; hsa-miR-6808-3p
positive regulation of endothelial cell proliferation (GO:0001938)	0.008397	NRAS;	hsa-miR-27b-3p; hsa-miR-145-5p; hsa-let-7a-5p; hsa-let-7c-5p; hsa-let-7i-5p; hsa-miR-6732-5p
positive regulation of endothelial cell proliferation (GO:0001938)	0.008397	BMPR2;	hsa-let-7a-5p; hsa-miR-143-3p
positive regulation of endothelial cell proliferation (GO:0001938)	0.008397	SP1;	hsa-let-7c-5p; hsa-let-7i-5p; hsa-miR-6732-5p
positive regulation of endothelial cell proliferation (GO:0001938)	0.008397	ITGB3;	hsa-let-7c-5p; hsa-miR-145-5p; hsa-let-7i-5p; hsa-let-7c-5p; hsa-let-7a-5p; hsa-miR-143-3p; hsa-miR-27b-3p
positive regulation of endothelial cell proliferation (GO:0001938)	0.008397	PDGFB;	hsa-let-7i-5p; hsa-miR-6808-3p; hsa-let-7a-5p; hsa-miR-27b-3p; hsa-miR-455-3p; hsa-miR-143-3p; hsa-miR-145-5p
positive regulation of endothelial cell proliferation (GO:0001938)	0.008397	NRARP;	hsa-miR-145-5p
positive regulation of endothelial cell proliferation (GO:0001938)	0.008397	TGFBR1	hsa-let-7a-5p; hsa-let-7c-5p; hsa-let-7i-5p; hsa-miR-6808-3p

Supplemental Table S2. Network of molecular pathways of the scaffold endothelialized with smooth muscle versus control.

Supplemental Table 2. Network of molecular pathways of the scaffold endothelialized with smooth muscle versus control.

Term	P-value	Genes	miRNA
Vascular system	0.00000000113	UBE2D3	hsa-miR-133a-3p;
Vascular system	0.00000000113	HNRNPU	hsa-miR-411-5p; hsa-miR-379-5p; hsa-miR-140-5p; hsa-miR-139-5p; hsa-miR-486-5p; hsa-miR-145-5p; hsa-miR-139-5p
Vascular system	0.00000000113	BZW1	hsa-miR-486-5p;
Vascular system	0.00000000113	MYLK	hsa-miR-139-5p; hsa-miR-411-5p; hsa-miR-133a-3p
Vascular system	0.00000000113	PPP1CB	hsa-miR-411-5p; hsa-miR-133a-3p; hsa-miR-145-5p; hsa-miR-139-5p
Vascular system	0.00000000113	CCND1	hsa-miR-497-5p
Vascular system	0.00000000113	CDH2	hsa-miR-411-5p; hsa-miR-145-5p; hsa-miR-497-5p
Vascular system	0.00000000113	LAMP2	hsa-miR-487b-3p; hsa-miR-145-5p; hsa-miR-140-5p; hsa-miR-133a-3p
Vascular system	0.00000000113	SLC38A1	hsa-miR-378c; hsa-miR-378f
Vascular system	0.00000000113	MBNL1	hsa-miR-126-3p; hsa-miR-486-5p; hsa-miR-411-5p; hsa-miR-145-5p; hsa-miR-139-5p
Vascular system	0.00000000113	FOXP1	hsa-miR-486-5p; hsa-miR-378c; hsa-miR-139-5p
Vascular system	0.00000000113	HNRNPUL1	hsa-miR-411-5p; hsa-miR-486-5p; hsa-miR-145-5p; hsa-miR-139-5p
Vascular system	0.00000000113	TMX1	hsa-miR-196b-5p
Vascular system	0.00000000113	TBL1XR1	hsa-miR-497-5p; hsa-miR-145-5p
Vascular system	0.00000000113	MAP1B	hsa-miR-532-5p; hsa-miR-411-5p; hsa-miR-145-5p
Vascular system	0.00000000113	PAPPA	hsa-miR-497-5p; hsa-miR-487b-3p; hsa-miR-378f; hsa-miR-378c
Vascular system	0.00000000113	SRSF2	hsa-miR-126-3p; hsa-miR-486-5p; hsa-miR-411-5p; hsa-miR-145-5p; hsa-miR-139-5p
Vascular system	0.00000000113	KCTD12	hsa-miR-497-5p; hsa-miR-487b-3p; hsa-miR-378f; hsa-miR-378c
Vascular system	0.00000000113	STC1	hsa-miR-497-5p; hsa-miR-487b-3p; hsa-miR-378f; hsa-miR-378c
Vascular system	0.00000000113	TWF1	hsa-miR-486-5p; hsa-miR-497-5p
Vascular system	0.00000000113	CORO1C	hsa-miR-133b; hsa-miR-133a-3p; hsa-miR-126-3p; hsa-miR-497-5p
Vascular system	0.00000000113	UBE2J1	hsa-miR-30e-3p; hsa-miR-487b-3p; hsa-miR-145-5p; hsa-miR-140-5p
Vascular system	0.00000000113	RAP1B	hsa-miR-497-5p; hsa-miR-133a-3p; hsa-miR-140-5p; hsa-miR-139-5p; hsa-miR-30e-3p
Vascular system	0.00000000113	RAP1A	hsa-miR-30e-3p; hsa-miR-486-5p
Vascular system	0.00000000113	SEC14L1	hsa-miR-30e-3p
Vascular system	0.00000000113	SSR1	hsa-miR-196b-5p
Vascular system	0.00000000113	RBM12	hsa-miR-486-5p
Vascular system	0.00000000113	SLC38A2	hsa-miR-378f; hsa-miR-145-5p; hsa-miR-140-5p
Vascular system	0.00000000113	SPTBN1	hsa-miR-487b-3p; hsa-miR-139-5p
Vascular system	0.00000000113	MCL1	hsa-miR-133a-3p; hsa-miR-497-5p
Vascular system	0.00000000113	CD164	hsa-miR-140-5p; hsa-miR-497-5p; hsa-miR-139-5p
Vascular system	0.00000000113	CAV1	hsa-miR-411-5p; hsa-miR-133a-3p
Vascular system	0.00000000113	SMARCA5	hsa-miR-379-5p; hsa-miR-145-5p
Vascular system	0.00000000113	CRIM1	hsa-miR-411-5p; hsa-miR-145-5p
Vascular system	0.00000000113	ARPC5	hsa-miR-497-5p
Vascular system	0.00000000113	AZIN1	hsa-miR-487b-3p; hsa-miR-139-5p
Vascular system	0.00000000113	CAPRIN1	hsa-miR-486-5p; hsa-miR-497-5p;
Vascular system	0.00000000113	CALU	hsa-miR-140-5p; hsa-miR-411-5p; hsa-miR-133a-3p

Vascular system	0.00000000113	TCF4	hsa-miR-497-5p; hsa-miR-145-5p; hsa-miR-497-5p
Vascular system	0.00000000113	CALM1	hsa-miR-411-5p; hsa-miR-133a-3p; hsa-miR-196b-5p; hsa-miR-497-5p; hsa-miR-133b
Vascular system	0.00000000113	EIF4G2	hsa-miR-411-5p; hsa-miR-379-5p; hsa-miR-140-5p; hsa-miR-139-5p
Vascular system	0.00000000113	YWHAE	hsa-miR-411-5p; hsa-miR-133a-3p; hsa-miR-30e-3p
Vascular system	0.00000000113	NRP1	hsa-miR-487b-3p
Vascular system	0.00000000113	BMPR2	hsa-miR-532-5p
Vascular system	0.00000000113	CLTC	hsa-miR-411-5p; hsa-miR-133a-3p; hsa-miR-497-5p; hsa-miR-145-5p
Vascular system	0.00000000113	LAMC1	hsa-miR-497-5p; hsa-miR-140-5p
Vascular system	0.00000000113	TRAM2	hsa-miR-3135b; hsa-miR-133b; hsa-miR-133a-3p
Vascular system	0.00000000113	TMEM47	hsa-miR-487b-3p; hsa-miR-145-5p; hsa-miR-140-5p
Vascular system	0.00000000113	XPO1	hsa-miR-140-5p; hsa-miR-139-5p; hsa-miR-532-5p; hsa-miR-411-5p; hsa-miR-486-5p; hsa-miR-378c; hsa-miR-145-5p; hsa-miR-497-5p; hsa-miR-133a-3p
Vascular system	0.00000000113	ENC1	hsa-miR-133a-3p; hsa-miR-497-5p; hsa-miR-133b
Vascular system	0.00000000113	TNPO1	hsa-miR-411-5p; hsa-miR-126-3p; hsa-miR-486-5p; hsa-miR-411-5p; hsa-miR-379-5p; hsa-miR-140-5p; hsa-miR-139-5p
Vascular system	0.00000000113	RAB8B	hsa-miR-204-3p; hsa-miR-411-5p; hsa-miR-133a-3p; hsa-miR-30e-3p; hsa-miR-497-5p; hsa-miR-126-3p
Vascular system	0.00000000113	CAP1	hsa-miR-497-5p; hsa-miR-133b
Vascular system	0.00000000113	ACTR2	hsa-miR-497-5p
Vascular system	0.00000000113	ENAH	hsa-miR-139-5p
Vascular system	0.00000000113	ATRN	hsa-miR-145-5p
Vascular system	0.00000000113	ZEB1	hsa-miR-139-5p; hsa-miR-497-5p; hsa-miR-411-5p; hsa-miR-145-5p
Vascular system	0.00000000113	ASPH	hsa-miR-126-3p
Vascular system	0.00000000113	SGK1	hsa-miR-133b; hsa-miR-497-5p; hsa-miR-133a-3p
Vascular system	0.00000000113	RAB1A	hsa-miR-204-3p; hsa-miR-497-5p; hsa-miR-486-5p; hsa-miR-411-5p
Vascular system	0.00000000113	RALA	hsa-miR-140-5p; hsa-miR-204-3p; hsa-miR-497-5p; hsa-miR-30e-3p; hsa-miR-411-5p; hsa-miR-133a-3p
Vascular system	0.00000000113	SRSF1	hsa-miR-486-5p; hsa-miR-139-5p; hsa-miR-145-5p; hsa-miR-140-5p; hsa-miR-126-3p; hsa-miR-411-5p
Vascular system	0.00000000113	GNAI3	hsa-miR-497-5p; hsa-miR-133a-3p
Vascular system	0.00000000113	THBS1	hsa-miR-497-5p; hsa-miR-140-5p; hsa-miR-139-5p
Vascular system	0.00000000113	CDC42	hsa-miR-411-5p; hsa-miR-133a-3p; hsa-miR-497-5p; hsa-miR-145-5p; hsa-miR-532-5p
Vascular system	0.00000000113	HNRNPA3	hsa-miR-486-5p; hsa-miR-411-5p; hsa-miR-411-5p; hsa-miR-379-5p; hsa-miR-140-5p; hsa-miR-139-5p
Vascular system	0.00000000113	TMEM30A	hsa-miR-378c; hsa-miR-411-5p; hsa-miR-204-3p
Vascular system	0.00000000113	GALNT1	hsa-miR-145-5p
Vascular system	0.00000000113	TXNRD1	hsa-miR-497-5p; hsa-miR-145-5p; hsa-miR-196b-5p; hsa-miR-30e-3p
Vascular system	0.00000000113	NAP1L1	hsa-miR-196b-5p; hsa-miR-379-5p; hsa-miR-145-5p
Vascular system	0.00000000113	RAB11A	hsa-miR-532-5p; hsa-miR-487b-3p; hsa-miR-145-5p; hsa-miR-140-5p; hsa-miR-204-3p; hsa-miR-411-5p; hsa-miR-145-5p
Vascular system	0.00000000113	RAB10	hsa-miR-140-5p; hsa-miR-378c; hsa-miR-378f; hsa-miR-497-5p
Vascular system	0.00000000113	UFM1	hsa-miR-497-5p
Vascular system	0.00000000113	HNRNPA2B1	hsa-miR-411-5p; hsa-miR-379-5p; hsa-miR-140-5p; hsa-miR-139-5p
Vascular system	0.00000000113	TARDBP	hsa-miR-126-3p; hsa-miR-486-5p; hsa-miR-411-5p; hsa-miR-145-5p; hsa-miR-139-5p

Vascular system	0.00000000113	CUL4B	hsa-miR-133a-3p
Muscle	1.40E-16	HNRNPU	hsa-miR-411-5p; hsa-miR-379-5p; hsa-miR-140-5p; hsa-miR-139-5p; hsa-miR-486-5p; hsa-miR-145-5p; hsa-miR-139-5p
Muscle	1.40E-16	MYLK	hsa-miR-139-5p; hsa-miR-411-5p; hsa-miR-133a-3p
Muscle	1.40E-16	MBNL1	hsa-miR-126-3p; hsa-miR-486-5p; hsa-miR-411-5p; hsa-miR-145-5p; hsa-miR-139-5p
Muscle	1.40E-16	SSR1	hsa-miR-196b-5p
Muscle	1.40E-16	RBM12	hsa-miR-486-5p
Muscle	1.40E-16	SPTBN1	hsa-miR-487b-3p; hsa-miR-139-5p
Muscle	1.40E-16	SMARCA5	hsa-miR-379-5p; hsa-miR-145-5p
Muscle	1.40E-16	CALU	hsa-miR-140-5p; hsa-miR-411-5p; hsa-miR-133a-3p
Muscle	1.40E-16	CALM1	hsa-miR-411-5p; hsa-miR-133a-3p; hsa-miR-196b-5p; hsa-miR-497-5p; hsa-miR-133b
Muscle	1.40E-16	NRP1	hsa-miR-487b-3p
Muscle	1.40E-16	CLTC	hsa-miR-411-5p; hsa-miR-133a-3p; hsa-miR-497-5p; hsa-miR-145-5p
Muscle	1.40E-16	XPO1	hsa-miR-140-5p; hsa-miR-139-5p; hsa-miR-532-5p; hsa-miR-411-5p; hsa-miR-486-5p; hsa-miR-378c; hsa-miR-145-5p; hsa-miR-497-5p; hsa-miR-133a-3p
Muscle	1.40E-16	ENC1	hsa-miR-133a-3p; hsa-miR-497-5p; hsa-miR-133b
Muscle	1.40E-16	RAB8B	hsa-miR-204-3p; hsa-miR-411-5p; hsa-miR-133a-3p; hsa-miR-30e-3p; hsa-miR-497-5p; hsa-miR-126-3p
Muscle	1.40E-16	ZEB1	hsa-miR-139-5p; hsa-miR-497-5p; hsa-miR-411-5p; hsa-miR-145-5p
Muscle	1.40E-16	SGK1	hsa-miR-133b; hsa-miR-497-5p; hsa-miR-133a-3p
Muscle	1.40E-16	BZW1	hsa-miR-486-5p;
Muscle	1.40E-16	PPP1CB	hsa-miR-411-5p; hsa-miR-133a-3p; hsa-miR-145-5p; hsa-miR-139-5p
Muscle	1.40E-16	SLC38A1	hsa-miR-378c; hsa-miR-378f
Muscle	1.40E-16	HNRNPUL1	hsa-miR-411-5p; hsa-miR-486-5p; hsa-miR-145-5p; hsa-miR-139-5p
Muscle	1.40E-16	TBL1XR1	hsa-miR-497-5p; hsa-miR-145-5p
Muscle	1.40E-16	SLC38A2	hsa-miR-378f; hsa-miR-145-5p; hsa-miR-140-5p
Muscle	1.40E-16	CD164	hsa-miR-140-5p; hsa-miR-497-5p; hsa-miR-139-5p
Muscle	1.40E-16	CAV1	hsa-miR-411-5p; hsa-miR-133a-3p
Muscle	1.40E-16	AZIN1	hsa-miR-487b-3p; hsa-miR-139-5p
Muscle	1.40E-16	CAPRIN1	hsa-miR-486-5p; hsa-miR-497-5p;
Muscle	1.40E-16	EIF4G2	hsa-miR-411-5p; hsa-miR-379-5p; hsa-miR-140-5p; hsa-miR-139-5p
Muscle	1.40E-16	YWHAE	hsa-miR-411-5p; hsa-miR-133a-3p; hsa-miR-30e-3p
Muscle	1.40E-16	BMPR2	hsa-miR-532-5p
Muscle	1.40E-16	LAMC1	hsa-miR-497-5p; hsa-miR-140-5p
Muscle	1.40E-16	TNPO1	hsa-miR-411-5p; hsa-miR-126-3p; hsa-miR-486-5p; hsa-miR-411-5p; hsa-miR-379-5p; hsa-miR-140-5p; hsa-miR-139-5p
Muscle	1.40E-16	ACTR2	hsa-miR-497-5p
Muscle	1.40E-16	ATRN	hsa-miR-145-5p
Muscle	1.40E-16	ASPH	hsa-miR-126-3p
Muscle	1.40E-16	THBS1	hsa-miR-497-5p; hsa-miR-140-5p; hsa-miR-139-5p
Muscle	1.40E-16	CDC42	hsa-miR-411-5p; hsa-miR-133a-3p; hsa-miR-497-5p; hsa-miR-145-5p; hsa-miR-532-5p
Muscle	1.40E-16	HNRNPA3	hsa-miR-486-5p; hsa-miR-411-5p; hsa-miR-411-5p; hsa-miR-379-5p; hsa-miR-140-5p; hsa-miR-139-5p
Muscle	1.40E-16	NAP1L1	hsa-miR-196b-5p; hsa-miR-379-5p; hsa-miR-145-5p

Muscle	1.40E-16	RAB10	hsa-miR-140-5p; hsa-miR-378c; hsa-miR-378f; hsa-miR-497-5p
Muscle	1.40E-16	CUL4B	hsa-miR-133a-3p
Smooth muscle	2.03E-16	MYLK	hsa-miR-139-5p; hsa-miR-411-5p; hsa-miR-133a-3p
Smooth muscle	2.03E-16	TMEM47	hsa-miR-487b-3p; hsa-miR-145-5p; hsa-miR-140-5p
Smooth muscle	2.03E-16	FOXP1	hsa-miR-486-5p; hsa-miR-378c; hsa-miR-139-5p
Smooth muscle	2.03E-16	SRSF2	hsa-miR-126-3p; hsa-miR-486-5p; hsa-miR-411-5p; hsa-miR-145-5p; hsa-miR-139-5p
Smooth muscle	2.03E-16	CORO1C	hsa-miR-133b; hsa-miR-133a-3p; hsa-miR-126-3p; hsa-miR-497-5p
Smooth muscle	2.03E-16	RAP1A	hsa-miR-30e-3p; hsa-miR-486-5p
Smooth muscle	2.03E-16	SLC38A2	hsa-miR-378f; hsa-miR-145-5p; hsa-miR-140-5p
Smooth muscle	2.03E-16	ARPC5	hsa-miR-497-5p
Smooth muscle	2.03E-16	ACTR2	hsa-miR-497-5p
Smooth muscle	2.03E-16	CDC42	hsa-miR-411-5p; hsa-miR-133a-3p; hsa-miR-497-5p; hsa-miR-145-5p; hsa-miR-532-5p
Smooth muscle	2.03E-16	HNRNPA3	hsa-miR-486-5p; hsa-miR-411-5p; hsa-miR-411-5p; hsa-miR-379-5p; hsa-miR-140-5p; hsa-miR-139-5p
Smooth muscle	2.03E-16	SEC14L1	hsa-miR-30e-3p
Smooth muscle	2.03E-16	RBM12	hsa-miR-486-5p
Smooth muscle	2.03E-16	SMARCA5	hsa-miR-379-5p; hsa-miR-145-5p
Smooth muscle	2.03E-16	CALU	hsa-miR-140-5p; hsa-miR-411-5p; hsa-miR-133a-3p
Smooth muscle	2.03E-16	CALM1	hsa-miR-411-5p; hsa-miR-133a-3p; hsa-miR-196b-5p; hsa-miR-497-5p; hsa-miR-133b
Smooth muscle	2.03E-16	NRP1	hsa-miR-487b-3p
Smooth muscle	2.03E-16	ENC1	hsa-miR-133a-3p; hsa-miR-497-5p; hsa-miR-133b
Smooth muscle	2.03E-16	HNRNPA2B1	hsa-miR-411-5p; hsa-miR-379-5p; hsa-miR-140-5p; hsa-miR-139-5p
Smooth muscle	2.03E-16	TARDBP	hsa-miR-126-3p; hsa-miR-486-5p; hsa-miR-411-5p; hsa-miR-145-5p; hsa-miR-139-5p
Smooth muscle	2.03E-16	CCND1	hsa-miR-497-5p
Smooth muscle	2.03E-16	HNRNPUL1	hsa-miR-411-5p; hsa-miR-486-5p; hsa-miR-145-5p; hsa-miR-139-5p
Smooth muscle	2.03E-16	CAV1	hsa-miR-411-5p; hsa-miR-133a-3p
Smooth muscle	2.03E-16	YWHAE	hsa-miR-411-5p; hsa-miR-133a-3p; hsa-miR-30e-3p
Smooth muscle	2.03E-16	LAMC1	hsa-miR-497-5p; hsa-miR-140-5p
Smooth muscle	2.03E-16	RALA	hsa-miR-140-5p; hsa-miR-204-3p; hsa-miR-497-5p; hsa-miR-30e-3p; hsa-miR-411-5p; hsa-miR-133a-3p
Smooth muscle	2.03E-16	GALNT1	hsa-miR-145-5p
Smooth muscle	2.03E-16	UFM1	hsa-miR-497-5p
Smooth muscle	2.03E-16	HNRNPU	hsa-miR-411-5p; hsa-miR-379-5p; hsa-miR-140-5p; hsa-miR-139-5p; hsa-miR-486-5p; hsa-miR-145-5p; hsa-miR-139-5p
Smooth muscle	2.03E-16	MBNL1	hsa-miR-126-3p; hsa-miR-486-5p; hsa-miR-411-5p; hsa-miR-145-5p; hsa-miR-139-5p
Smooth muscle	2.03E-16	MAP1B	hsa-miR-532-5p; hsa-miR-411-5p; hsa-miR-145-5p
Smooth muscle	2.03E-16	SSR1	hsa-miR-196b-5p
Smooth muscle	2.03E-16	ZEB1	hsa-miR-139-5p; hsa-miR-497-5p; hsa-miR-411-5p; hsa-miR-145-5p
Smooth muscle	2.03E-16	PPP1CB	hsa-miR-411-5p; hsa-miR-133a-3p; hsa-miR-145-5p; hsa-miR-139-5p
Smooth muscle	2.03E-16	CDH2	hsa-miR-411-5p; hsa-miR-145-5p; hsa-miR-497-5p
Smooth muscle	2.03E-16	LAMP2	hsa-miR-487b-3p; hsa-miR-145-5p; hsa-miR-140-5p; hsa-miR-133a-3p
Smooth muscle	2.03E-16	MCL1	hsa-miR-133a-3p; hsa-miR-497-5p
Smooth muscle	2.03E-16	CD164	hsa-miR-140-5p; hsa-miR-497-5p; hsa-miR-139-5p
Smooth muscle	2.03E-16	EIF4G2	hsa-miR-411-5p; hsa-miR-379-5p; hsa-miR-140-5p; hsa-miR-139-5p

Smooth muscle	2.03E-16	TNPO1	hsa-miR-411-5p; hsa-miR-126-3p; hsa-miR-486-5p; hsa-miR-411-5p; hsa-miR-379-5p; hsa-miR-140-5p; hsa-miR-139-5p
Smooth muscle	2.03E-16	ASPH	hsa-miR-126-3p
Smooth muscle	2.03E-16	RAB1A	hsa-miR-204-3p; hsa-miR-497-5p; hsa-miR-486-5p; hsa-miR-411-5p
Smooth muscle	2.03E-16	TXNRD1	hsa-miR-497-5p; hsa-miR-145-5p; hsa-miR-196b-5p; hsa-miR-30e-3p
Smooth muscle	2.03E-16	RAB10	hsa-miR-140-5p; hsa-miR-378c; hsa-miR-378f; hsa-miR-497-5p
Smooth muscle	2.03E-16	CUL4B	hsa-miR-133a-3p
Smooth muscle	2.03E-16	STC1	hsa-miR-497-5p; hsa-miR-487b-3p; hsa-miR-378f; hsa-miR-378c
Smooth muscle	2.03E-16	TWF1	hsa-miR-486-5p; hsa-miR-497-5p
Smooth muscle	2.03E-16	TRAM2	hsa-miR-3135b; hsa-miR-133b; hsa-miR-133a-3p
Smooth muscle	2.03E-16	XPO1	hsa-miR-140-5p; hsa-miR-139-5p; hsa-miR-532-5p; hsa-miR-411-5p; hsa-miR-486-5p; hsa-miR-378c; hsa-miR-145-5p; hsa-miR-497-5p; hsa-miR-133a-3p
Smooth muscle	2.03E-16	SGK1	hsa-miR-133b; hsa-miR-497-5p; hsa-miR-133a-3p
Smooth muscle	2.03E-16	RAB11A	hsa-miR-532-5p; hsa-miR-487b-3p; hsa-miR-145-5p; hsa-miR-140-5p; hsa-miR-204-3p; hsa-miR-411-5p; hsa-miR-145-5p
Smooth muscle	2.03E-16	TBL1XR1	hsa-miR-497-5p; hsa-miR-145-5p
Smooth muscle	2.03E-16	AZIN1	hsa-miR-487b-3p; hsa-miR-139-5p
Smooth muscle	2.03E-16	CAPRIN1	hsa-miR-486-5p; hsa-miR-497-5p;
Smooth muscle	2.03E-16	THBS1	hsa-miR-497-5p; hsa-miR-140-5p; hsa-miR-139-5p
Smooth muscle	2.03E-16	TMEM30A	hsa-miR-378c; hsa-miR-411-5p; hsa-miR-204-3p
Smooth muscle	2.03E-16	NAP1L1	hsa-miR-196b-5p; hsa-miR-379-5p; hsa-miR-145-5p
VEGF activated receptor activity (GO:0005021)	0.008489151	NRP1	hsa-miR-487b-3p
endothelial cell development (GO:0001885)	0.006351	RAP1B	hsa-miR-497-5p; hsa-miR-133a-3p; hsa-miR-140-5p; hsa-miR-139-5p; hsa-miR-30e-3p
endothelial cell development (GO:0001885)	0.006351	RAP1A	hsa-miR-30e-3p; hsa-miR-486-5p
endothelial cell development (GO:0001885)	0.006351	STC1	hsa-miR-497-5p; hsa-miR-487b-3p; hsa-miR-378f; hsa-miR-378c
regulation of angiogenesis (GO:0045765)	0.009138	THBS1	hsa-miR-497-5p; hsa-miR-140-5p; hsa-miR-139-5p

Supplemental Table S3. Molecular pathway network of endothelial cells versus undifferentiated MSC

Supplemental Table 3. Molecular pathway network of endothelial cells versus undifferentiated MSC

Term	P-value	Genes	miRNA
Negative Regulation Of Cell Differentiation (GO:0045596)	6.26E-06	JAG1;	hsa-miR-151a-5p; hsa-miR-8089; hsa-miR-4758-5p; hsa-miR-191-5p; hsa-miR-26a-5p; hsa-miR-4433b-3p; hsa-miR-199a-5p
Negative Regulation Of Cell Differentiation (GO:0045596)	6.26E-06	YTHDF2;	hsa-miR-6125; hsa-miR-1227-5p; hsa-miR-4433b-3p; hsa-miR-145-5p; hsa-miR-99b-5p
Negative Regulation Of Cell Differentiation (GO:0045596)	6.26E-06	SMAD3;	hsa-miR-3135b; hsa-miR-4508; hsa-miR-1227-5p; hsa-miR-151a-5p; hsa-miR-4270; hsa-miR-4758-5p; hsa-miR-6722-3p ; hsa-miR-4433b-3p; hsa-miR-145-5p; hsa-miR-4505; hsa-miR-3196; hsa-miR-3940-5p
Negative Regulation Of Cell Differentiation (GO:0045596)	6.26E-06	SEMA4D;	hsa-miR-4463; hsa-miR-125a-5p; hsa-miR-1225-5p
Negative Regulation Of Cell Differentiation (GO:0045596)	6.26E-06	PTEN;	hsa-miR-3135b; hsa-miR-151a-5p; hsa-miR-4270; hsa-miR-8089; hsa-miR-4758-5p; hsa-miR-191-5p; hsa-miR-26a-5p; hsa-miR-92a-3p; hsa-miR-29a-3p; hsa-miR-214-3p; hsa-miR-320d; hsa-miR-320c; hsa-miR-320b; hsa-miR-152-3p
Negative Regulation Of Cell Differentiation (GO:0045596)	6.26E-06	DLL1;	hsa-miR-107; hsa-miR-15b-5p
Negative Regulation Of Cell Differentiation (GO:0045596)	6.26E-06	TGFBR1;	hsa-miR-3135b; hsa-miR-4758-5p; hsa-miR-4433b-3p; hsa-let-7a-5p; hsa-let-7d-5p; hsa-let-7c-5p
Negative Regulation Of Cell Differentiation (GO:0045596)	6.26E-06	SMAD7;	hsa-miR-3135b
Negative Regulation Of Cell Differentiation (GO:0045596)	6.26E-06	COL5A1;	hsa-miR-6727-5p ; hsa-miR-3196; hsa-miR-3178; hsa-miR-29a-3p; hsa-miR-4734
Negative Regulation Of Cell Differentiation (GO:0045596)	6.26E-06	MYB;	hsa-miR-107; hsa-miR-15b-5p
Negative Regulation Of Cell Differentiation (GO:0045596)	6.26E-06	COL5A2;	hsa-let-7d-5p; hsa-miR-29a-3p
Negative Regulation Of Cell Differentiation (GO:0045596)	6.26E-06	PDCD4;	hsa-miR-4270; hsa-miR-145-5p; hsa-miR-15b-5p
Negative Regulation Of Cell Differentiation (GO:0045596)	6.26E-06	SPRY2;	hsa-miR-3135b; hsa-miR-8089; hsa-miR-4758-5p; hsa-miR-191-5p; hsa-miR-4433b-3p; hsa-miR-27b-3p
Negative Regulation Of Cell Differentiation (GO:0045596)	6.26E-06	ITGAV;	hsa-miR-151a-5p; hsa-miR-4758-5p; hsa-miR-92a-3p
Negative Regulation Of Cell Differentiation (GO:0045596)	6.26E-06	RUNX1T1	hsa-miR-4739; hsa-miR-4270; hsa-miR-107; hsa-miR-15b-5p
Positive Regulation Of Cell Differentiation (GO:0045597)	1.26E-04	ACVR1;	hsa-miR-152-3p; hsa-miR-1225-5p
Positive Regulation Of Cell Differentiation (GO:0045597)	1.26E-04	SMAD3;	hsa-miR-3135b; hsa-miR-4508; hsa-miR-1227-5p; hsa-miR-151a-5p; hsa-miR-4270; hsa-miR-4758-5p; hsa-miR-6722-3p; hsa-miR-4433b-3p; hsa-miR-145-5p; hsa-miR-4505; hsa-miR-3196 ; hsa-miR-3940-5p
Positive Regulation Of Cell Differentiation (GO:0045597)	1.26E-04	MMD;	
Positive Regulation Of Cell Differentiation (GO:0045597)	1.26E-04	SOX11;	hsa-miR-4739; hsa-miR-151a-5p ; hsa-miR-221-3p; hsa-miR-145-5p; hsa-miR-6727-5p; hsa-miR-3178; hsa-miR-92a-3p; hsa-miR-27b-3p; hsa-miR-125a-5p; hsa-miR-214-3p; hsa-miR-149-3p; hsa-miR-152-3p
Positive Regulation Of Cell Differentiation (GO:0045597)	1.26E-04	AXIN2;	hsa-miR-222-3p; hsa-miR-107; hsa-miR-15b-5p
Positive Regulation Of Cell Differentiation (GO:0045597)	1.26E-04	ACVR2B;	hsa-miR-99b-5p; hsa-miR-99a-5p; hsa-miR-937-5p; hsa-miR-6819-5p; hsa-miR-6765-5p; hsa-miR-574-3p; hsa-miR-574-3p; hsa-miR-199a-5p; hsa-miR-107; hsa-miR-199b-3p; hsa-miR-199a-3p; hsa-miR-181b-5p; hsa-miR-181a-5p; hsa-miR-15b-5p
Positive Regulation Of Cell Differentiation (GO:0045597)	1.26E-04	TGFBR1;	hsa-miR-3135b; hsa-miR-4758-5p; hsa-miR-4433b-3p; hsa-let-7a-5p; hsa-let-7d-5p; hsa-let-7c-5p
Positive Regulation Of Cell Differentiation (GO:0045597)	1.26E-04	ACVR2A;	hsa-miR-3940-5p; hsa-miR-199b-3p;hsa-miR-3940-5p; hsa-miR-199b-3p; hsa-miR-199a-3p; hsa-miR-181b-5p; hsa-miR-181a-5p; hsa-miR-15b-5p; hsa-let-7c-5p
Positive Regulation Of Cell Differentiation (GO:0045597)	1.26E-04	XKR8;	hsa-miR-199a-3p; hsa-miR-181b-5p; hsa-miR-181a-5p; hsa-miR-15b-5p; hsa-let-7c-5p
Positive Regulation Of Cell Differentiation (GO:0045597)	1.26E-04	RPS6KA3;	hsa-miR-6802-5p; hsa-miR-3135b; hsa-miR-8089; hsa-miR-4758-5p; hsa-miR-4298; hsa-miR-4516; hsa-miR-107; hsa-miR-15b-5p ; hsa-miR-152-3p; hsa-miR-1225-5p
Positive Regulation Of Cell Differentiation (GO:0045597)	1.26E-04	PPP3CA;	hsa-miR-145-5p; hsa-miR-100-5p; hsa-miR-99b-5p; hsa-miR-99a-5p
Positive Regulation Of Cell Differentiation (GO:0045597)	1.26E-04	SDCBP;	hsa-miR-151a-5p; hsa-miR-107
Positive Regulation Of Cell Differentiation (GO:0045597)	1.26E-04	DAB2;	hsa-miR-145-5p; hsa-miR-197-5p

Positive Regulation Of Cell Differentiation (GO:0045597)	1.26E-04	RBM24;	hsa-miR-1227-5p ; hsa-miR-4687-3p;hsa-miR-221-3p; hsa-miR-222-3p; hsa-miR-320d; hsa-miR-320c; hsa-miR-320b; hsa-miR-107 ; hsa-miR-152-3p
Positive Regulation Of Cell Differentiation (GO:0045597)	1.26E-04	FGFR1	hsa-miR-8089; hsa-miR-4758-5p; hsa-miR-191-5p; hsa-miR-6727-5p; hsa-miR-214-3p; hsa-miR-15b-5p
Regulation Of Cell Adhesion (GO:0030155)	3.22E-04	PPP1CB;	hsa-miR-3135b; hsa-miR-4508; hsa-miR-191-5p; hsa-miR-8069; hsa-miR-100-5p; hsa-miR-99b-5p; hsa-miR-99a-5p; hsa-miR-181a-5p; hsa-miR-152-3p
Regulation Of Cell Adhesion (GO:0030155)	3.22E-04	PPP3CA;	hsa-miR-145-5p; hsa-miR-100-5p; hsa-miR-99b-5p; hsa-miR-99a-5p
Regulation Of Cell Adhesion (GO:0030155)	3.22E-04	NUAK1;	hsa-miR-4508; hsa-miR-4687-3p; hsa-miR-145-5p; hsa-miR-214-3p
Regulation Of Cell Adhesion (GO:0030155)	3.22E-04	YTHDF2;	hsa-miR-6125; hsa-miR-1227-5p; hsa-miR-4433b-3p; hsa-miR-145-5p; hsa-miR-99b-5p
Regulation Of Cell Adhesion (GO:0030155)	3.22E-04	ROCK1;	hsa-miR-3135b; hsa-miR-152-3p
Regulation Of Cell Adhesion (GO:0030155)	3.22E-04	SEMA4D;	hsa-miR-4463; hsa-miR-125a-5p; hsa-miR-1225-5p
Regulation Of Cell Adhesion (GO:0030155)	3.22E-04	PDE3B;	hsa-miR-6722-3p; hsa-miR-4433b-3p; hsa-miR-107
Regulation Of Cell Adhesion (GO:0030155)	3.22E-04	ITGAV;	hsa-miR-151a-5p; hsa-miR-4758-5p; hsa-miR-92a-3p
Regulation Of Cell Adhesion (GO:0030155)	3.22E-04	TNPO1;	hsa-miR-4687-3p; hsa-miR-26a-5p; hsa-miR-4433b-3p ; hsa-miR-27b-3p; hsa-miR-214-3p; hsa-miR-107; hsa-miR-181b-5p; hsa-miR-181a-5p
Regulation Of Cell Adhesion (GO:0030155)	3.22E-04	CELSR2;	hsa-miR-6775-5p; hsa-miR-4651; hsa-miR-4649-5p; hsa-miR-4505; hsa-miR-3196; hsa-miR-3178; hsa-miR-8069; hsa-miR-214-3p; hsa-miR-214-3p; hsa-miR-107; hsa-miR-199b-3p; hsa-miR-199a-3p
Regulation Of Cell Adhesion (GO:0030155)	3.22E-04	DLL1	hsa-miR-107; hsa-miR-15b-5p
Fibroblast Growth Factor Receptor Signaling Pathway (GO:0008543)	4.22E-04	TRIM71;	hsa-miR-1227-5p; hsa-miR-4270
Fibroblast Growth Factor Receptor Signaling Pathway (GO:0008543)	4.22E-04	RAB14;	hsa-miR-4739; hsa-miR-145-5p; hsa-miR-92a-3p; hsa-miR-214-3p; hsa-miR-152-3p
Fibroblast Growth Factor Receptor Signaling Pathway (GO:0008543)	4.22E-04	FLRT3;	hsa-miR-4758-5p; hsa-miR-4463; hsa-miR-320d; hsa-miR-199a-5p; hsa-miR-199b-3p
Fibroblast Growth Factor Receptor Signaling Pathway (GO:0008543)	4.22E-04	SHOC2;	hsa-miR-3135b; hsa-miR-199a-5p; hsa-miR-15b-5p
Fibroblast Growth Factor Receptor Signaling Pathway (GO:0008543)	4.22E-04	FGF2;	hsa-miR-4758-5p; hsa-miR-191-5p; hsa-miR-4463; hsa-miR-107; hsa-miR-15b-5p; hsa-miR-1225-5p
Fibroblast Growth Factor Receptor Signaling Pathway (GO:0008543)	4.22E-04	FGFR1	hsa-miR-8089; hsa-miR-4758-5p; hsa-miR-191-5p; hsa-miR-6727-5p; hsa-miR-214-3p; hsa-miR-15b-5p
Positive Regulation Of Sprouting Angiogenesis (GO:1903672)	4.53E-04	ITGA5;	hsa-miR-92a-3p; hsa-miR-152-3p
Positive Regulation Of Sprouting Angiogenesis (GO:1903672)	4.53E-04	KLF4;	hsa-miR-4758-5p; hsa-miR-92a-3p; hsa-miR-152-3p
Positive Regulation Of Sprouting Angiogenesis (GO:1903672)	4.53E-04	DLL1;	hsa-miR-107; hsa-miR-15b-5p
Positive Regulation Of Sprouting Angiogenesis (GO:1903672)	4.53E-04	FGF2	hsa-miR-4758-5p; hsa-miR-191-5p; hsa-miR-4463; hsa-miR-107; hsa-miR-15b-5p; hsa-miR-1225-5p
Regulation Of Vascular Endothelial Cell Proliferation (GO:1905562)	9.55E-04	FAM98A;	hsa-miR-26a-5p
Regulation Of Vascular Endothelial Growth Factor Signaling Pathway (GO:1900746)	0.001185614	EIF4G2;	hsa-miR-26a-5p; hsa-miR-4433b-3p; hsa-let-7a-5p; hsa-let-7d-5p; hsa-let-7c-5p
Regulation Of Sprouting Angiogenesis (GO:1903670)	0.001378468	SEMA6A;	hsa-miR-7847-3p; hsa-miR-145-5p; hsa-miR-27b-3p ; hsa-miR-1225-5p
Regulation Of Sprouting Angiogenesis (GO:1903670)	0.001378468	ITGA5;	hsa-miR-92a-3p; hsa-miR-152-3p
Regulation Of Sprouting Angiogenesis (GO:1903670)	0.001378468	KLF4;	hsa-miR-4758-5p; hsa-miR-92a-3p; hsa-miR-152-3p
Regulation Of Sprouting Angiogenesis (GO:1903670)	0.001378468	DLL1;	hsa-miR-107; hsa-miR-15b-5p
Regulation Of Sprouting Angiogenesis (GO:1903670)	0.001378468	FGF2	hsa-miR-4758-5p; hsa-miR-191-5p; hsa-miR-4463; hsa-miR-107; hsa-miR-15b-5p; hsa-miR-1225-5p
Cellular Response To Fibroblast Growth Factor Stimulus (GO:0044344)	0.002660601	TRIM71;	hsa-miR-1227-5p; hsa-miR-4270

Cellular Response To Fibroblast Growth Factor Stimulus (GO:0044344)	0.002660601	RAB14;	hsa-miR-4739; hsa-miR-145-5p; hsa-miR-92a-3p; hsa-miR-214-3p ; hsa-miR-152-3p
Cellular Response To Fibroblast Growth Factor Stimulus (GO:0044344)	0.002660601	FLRT3;	hsa-miR-4758-5p; hsa-miR-4463; hsa-miR-320d; hsa-miR-199a-5p; hsa-miR-199b-3p
Cellular Response To Fibroblast Growth Factor Stimulus (GO:0044344)	0.002660601	SHOC2;	hsa-miR-3135b; hsa-miR-199a-5p; hsa-miR-15b-5p
Cellular Response To Fibroblast Growth Factor Stimulus (GO:0044344)	0.002660601	FGF2;	hsa-miR-4758-5p; hsa-miR-191-5p; hsa-miR-4463; hsa-miR-107; hsa-miR-15b-5p; hsa-miR-1225-5p
Cellular Response To Fibroblast Growth Factor Stimulus (GO:0044344)	0.002660601	FGFR1	hsa-miR-8089; hsa-miR-4758-5p; hsa-miR-191-5p; hsa-miR-6727-5p; hsa-miR-214-3p; hsa-miR-15b-5p
Positive Regulation Of Vascular Endothelial Cell Proliferation (GO:1905564)	0.003037184	AKT3;	hsa-miR-320d; hsa-miR-320c; hsa-miR-320b; hsa-miR-181b-5p; hsa-miR-181a-5p; hsa-miR-15b-5p
Positive Regulation Of Vascular Endothelial Cell Proliferation (GO:1905564)	0.003037184	FGF2;	hsa-miR-4758-5p; hsa-miR-191-5p; hsa-miR-4463; hsa-miR-107; hsa-miR-15b-5p; hsa-miR-1225-5p
Positive Regulation Of Vascular Endothelial Cell Proliferation (GO:1905564)	0.003037184	FGFR1	hsa-miR-8089; hsa-miR-4758-5p; hsa-miR-191-5p; hsa-miR-6727-5p; hsa-miR-214-3p; hsa-miR-15b-5p

Supplemental Table S4. Molecular pathway network of smooth muscle cells versus undifferentiated MSCs.

Supplemental Table 4. Molecular pathway network of smooth muscle cells versus undifferentiated MSCs.

Term	P-value	Genes	miRNA
SmoothMuscle	5.00E-12	PID1	hsa-miR-23a-5p; hsa-miR-6870-5p
SmoothMuscle	5.00E-12	SERPINE1	hsa-miR-6165
SmoothMuscle	5.00E-12	HDLBP	hsa-miR-502-3p
SmoothMuscle	5.00E-12	PLOD1	hsa-miR-500a-3p; hsa-miR-3663-3p
SmoothMuscle	5.00E-12	FGF2	hsa-miR-4685-5p; hsa-miR-296-3p; hsa-miR-1224-5p ; hsa-miR-557; hsa-miR-502-3p; hsa-miR-615-5p ; hsa-miR-615-5p; hsa-miR-184; hsa-miR-3679-5p
SmoothMuscle	5.00E-12	LOXL2;	hsa-miR-296-3p
SmoothMuscle	5.00E-12	CDH6;	hsa-miR-221-3p
SmoothMuscle	5.00E-12	LACTB;	hsa-miR-615-5p; hsa-miR-574-3p
SmoothMuscle	5.00E-12	POFUT2;	hsa-miR-197-5p; hsa-miR-6870-5p; hsa-miR-34a-3p; hsa-miR-23b-5p; hsa-miR-3679-5p
SmoothMuscle	5.00E-12	TUBB6;	hsa-miR-6741-5p; hsa-miR-6889-5p
SmoothMuscle	5.00E-12	ALCAM;	hsa-miR-181a-5p; hsa-let-7i-5p; hsa-miR-222-3p; hsa-miR-143-3p; hsa-miR-421; hsa-miR-708-5p; hsa-miR-214-5p; hsa-miR-296-3p; hsa-miR-1909-3p
SmoothMuscle	5.00E-12	TFG;	hsa-miR-1229-5p; hsa-miR-504-3p
SmoothMuscle	5.00E-12	CDH2;	hsa-miR-222-3p; hsa-miR-143-3p; hsa-miR-221-3p; hsa-miR-328-3p; hsa-miR-145-5p; hsa-miR-502-3p; hsa-miR-501-3p; hsa-miR-421; hsa-miR-421; hsa-miR-708-5p; hsa-miR-1182; hsa-miR-296-3p; hsa-miR-1909-3p; hsa-miR-3679-5p
SmoothMuscle	5.00E-12	PLS3;	hsa-miR-6860; hsa-miR-6870-5p
SmoothMuscle	5.00E-12	PTGFRN;	hsa-miR-6846-5p; hsa-miR-1587; hsa-miR-933; hsa-miR-214-5p; hsa-miR-99a-5p
SmoothMuscle	5.00E-12	POSTN;	hsa-miR-6870-5p
SmoothMuscle	5.00E-12	IGFBP4;	hsa-miR-502-3p
SmoothMuscle	5.00E-12	ACTN1;	hsa-miR-34a-5p; hsa-miR-195-5p; hsa-miR-501-3p; hsa-miR-421; hsa-miR-222-3p; hsa-miR-874-3p; hsa-miR-1909-3p; hsa-miR-557; hsa-miR-1224-5p; hsa-miR-214-5p; hsa-miR-296-3p; hsa-miR-1909-3p
SmoothMuscle	5.00E-12	APLP2;	hsa-let-7i-5p; hsa-miR-214-5p; hsa-miR-4269; hsa-miR-1275
SmoothMuscle	5.00E-12	FNDC3B;	hsa-miR-1275; hsa-miR-328-3p; hsa-miR-1224-5p; hsa-miR-502-3p; hsa-miR-146a-5p; hsa-miR-708-5p
SmoothMuscle	5.00E-12	PDIA6;	hsa-miR-3663-3p
SmoothMuscle	5.00E-12	ADAM19;	hsa-miR-34a-5p; hsa-miR-195-5p
SmoothMuscle	5.00E-12	RAB32;	hsa-miR-1587
SmoothMuscle	5.00E-12	LOX;	hsa-miR-328-3p
SmoothMuscle	5.00E-12	KDELR2;	hsa-miR-874-3p
SmoothMuscle	5.00E-12	IL6ST;	hsa-miR-150-3p; hsa-miR-328-3p; hsa-miR-502-3p; hsa-miR-184
SmoothMuscle	5.00E-12	RAI14;	hsa-miR-23b-5p; hsa-miR-3679-5p
SmoothMuscle	5.00E-12	TTYH3;	hsa-miR-6742-5p; hsa-miR-6889-5p; hsa-miR-6824-5p
SmoothMuscle	5.00E-12	SAR1A;	hsa-miR-23a-5p; hsa-miR-3679-5p

SmoothMuscle	5.00E-12	FAM114A1;	hsa-miR-615-5p
SmoothMuscle	5.00E-12	LYPD1;	hsa-miR-3135b
SmoothMuscle	5.00E-12	TWF1;	hsa-miR-933
SmoothMuscle	5.00E-12	HSP90B1;	hsa-miR-615-5p; hsa-miR-296-3; hsa-miR-874-3p; hsa-miR-501-3p; hsa-miR-328-3p; hsa-miR-502-3p; hsa-miR-1275
SmoothMuscle	5.00E-12	FUT8;	hsa-miR-328-3p
SmoothMuscle	5.00E-12	CALD1;	hsa-miR-181a-5p; hsa-miR-145-5p; hsa-miR-181a-5p; hsa-let-7i-5p; hsa-miR-328-3p; hsa-miR-143-3p; hsa-miR-328-3p; hsa-miR-145-5p; hsa-miR-502-3p; hsa-miR-221-3p; hsa-miR-501-3p; hsa-miR-421; hsa-miR-222-3p; hsa-miR-557; hsa-miR-1224-5p; hsa-miR-146a-5p; hsa-miR-214-5p; hsa-miR-1275; hsa-miR-296-3p
SmoothMuscle	5.00E-12	STC2;	hsa-miR-504-3p; hsa-miR-6165; hsa-miR-99a-5p
SmoothMuscle	5.00E-12	ABL1;	hsa-miR-195-5p; hsa-miR-181a-5p; hsa-let-7i-5p; hsa-miR-222-3p; hsa-miR-143-3p; hsa-miR-501-3p; hsa-miR-421; hsa-miR-222-3p; hsa-miR-874-3p; hsa-miR-328-3p
SmoothMuscle	5.00E-12	PGK1;	hsa-miR-1587; hsa-miR-3180-3p;hsa-miR-6870-5p
SmoothMuscle	5.00E-12	ST3GAL1;	hsa-miR-6165
SmoothMuscle	5.00E-12	MYH10;	hsa-miR-34a-3p; hsa-miR-99a-5p; hsa-miR-197-5p
SmoothMuscle	5.00E-12	SLC38A2;	hsa-miR-6860; hsa-miR-23a-5p; hsa-miR-3180-3p; hsa-miR-504-3p
SmoothMuscle	5.00E-12	SOCS5;	hsa-miR-6165
SmoothMuscle	5.00E-12	ABCA1;	hsa-miR-34a-5p; hsa-miR-195-5p; hsa-miR-214-5p; hsa-miR-1275
SmoothMuscle	5.00E-12	TGFB2;	hsa-miR-6742-5p
SmoothMuscle	5.00E-12	SMAD3;	hsa-miR-6846-5p; hsa-miR-933
SmoothMuscle	5.00E-12	SMURF2;	hsa-miR-23a-5p
SmoothMuscle	5.00E-12	BDNF;	hsa-miR-181a-5p; hsa-let-7i-5p; hsa-miR-222-3p; hsa-miR-143-3p; hsa-miR-501-3p; hsa-miR-421; hsa-miR-222-3p; hsa-miR-421; hsa-miR-708-5p; hsa-miR-184; hsa-miR-1275
SmoothMuscle	5.00E-12	GFPT2;	hsa-miR-3679-5p; hsa-miR-501-3p; hsa-miR-214-5p
SmoothMuscle	5.00E-12	TMEM132A;	hsa-miR-6741-5p
SmoothMuscle	5.00E-12	GFPT1;	miR-3679-5p; hsa-miR-501-3p; hsa-miR-708-5p; hsa-miR-1275; hsa-miR-214-5p
SmoothMuscle	5.00E-12	FN1;	hsa-miR-146a-5p; hsa-miR-557; hsa-miR-1182; hsa-miR-502-3p; hsa-miR-214-5p; hsa-miR-1909-3p; hsa-miR-296-3p; hsa-miR-615-5p; hsa-miR-184; hsa-miR-1275; hsa-miR-34a-3p; hsa-miR-99a-5p; hsa-miR-3679-5p; hsa-miR-197-5p
SmoothMuscle	5.00E-12	MICAL2;	hsa-miR-4269; hsa-miR-4685-5p
SmoothMuscle	5.00E-12	ARHGAP29;	hsa-miR-221-3p
SmoothMuscle	5.00E-12	INHBA;	hsa-miR-1182; hsa-miR-197-5p
SmoothMuscle	5.00E-12	FOSL2;	hsa-miR-3135b; hsa-miR-502-3p; hsa-miR-146a-5p; hsa-miR-184
SmoothMuscle	5.00E-12	COL1A1;	hsa-miR-328-3p; hsa-miR-143-3p; hsa-miR-145-5p; hsa-miR-502-3p; hsa-miR-221-3p; hsa-miR-222-3p; hsa-miR-421; hsa-miR-708-5p; hsa-miR-557; hsa-miR-1224-5p; hsa-miR-146a-5p; hsa-miR-4685-5p; hsa-miR-296-3p; hsa-miR-1224-5p; hsa-miR-1182; hsa-miR-214-5p; hsa-miR-296-3p; hsa-miR-1909-3p
SmoothMuscle	5.00E-12	IL6;	hsa-miR-150-3p; hsa-miR-328-3p; hsa-miR-502-3p; hsa-miR-615-5p; hsa-miR-184
SmoothMuscle	5.00E-12	COL1A2;	hsa-miR-328-3p; hsa-miR-143-3p; hsa-miR-145-5p; hsa-miR-502-3p; hsa-miR-221-3p; hsa-miR-222-3p; hsa-miR-421; hsa-miR-708-5p; hsa-miR-146a-5p; hsa-miR-1182; hsa-miR-214-5p; hsa-miR-296-3p; hsa-miR-1909-3p
SmoothMuscle	5.00E-12	P4HA1;	hsa-miR-3679-5p

SmoothMuscle	5.00E-12	COL5A1;	hsa-miR-328-3p; hsa-miR-143-3p; hsa-miR-145-5p; hsa-miR-502-3p; hsa-miR-222-3p; hsa-miR-557; hsa-miR-1224-5p; hsa-miR-1275; hsa-miR-146a-5p; hsa-miR-708-5p; hsa-miR-214-5p; hsa-miR-296-3p; hsa-miR-1909-3p
SmoothMuscle	5.00E-12	P4HA2;	hsa-miR-3663-3p
SmoothMuscle	5.00E-12	RPL27A;	hsa-miR-6824-5p
SmoothMuscle	5.00E-12	COL5A2;	hsa-miR-328-3p; hsa-miR-143-3p; hsa-miR-145-5p; hsa-miR-502-3p; hsa-miR-557; hsa-miR-1224-5p; hsa-miR-146a-5p; hsa-miR-214-5p; hsa-miR-296-3p; hsa-miR-1909-3p
SmoothMuscle	5.00E-12	CALU;	hsa-miR-181a-5p; hsa-miR-708-5p; hsa-miR-501-3p; hsa-miR-328-3p
SmoothMuscle	5.00E-12	NF2;	hsa-miR-3180-3p; hsa-miR-184; hsa-miR-4269; hsa-miR-214-5p; hsa-miR-3135b
SmoothMuscle	5.00E-12	FERMT2;	hsa-miR-1909-3p; hsa-miR-557; hsa-miR-1224-5p; hsa-miR-1909-3p; hsa-miR-296-3p; hsa-miR-615-5p ; hsa-miR-615-5p; hsa-miR-1275
SmoothMuscle	5.00E-12	LRP12;	hsa-miR-1275; hsa-miR-3135b; hsa-miR-615-5p
SmoothMuscle	5.00E-12	FBN1;	hsa-miR-502-3p; hsa-miR-146a-5p; hsa-miR-1909-3p; hsa-miR-296-3p; hsa-miR-615-5p
SmoothMuscle	5.00E-12	ITGB1;	hsa-miR-214-5p; hsa-miR-615-5p; hsa-miR-1275; hsa-miR-34a-3p
SmoothMuscle	5.00E-12	ARF4;	hsa-miR-222-3; hsa-miR-143-3p; hsa-miR-34a-5p; hsa-miR-874-3p
SmoothMuscle	5.00E-12	NRP1;	hsa-miR-99a-5p; hsa-miR-3157-3p
SmoothMuscle	5.00E-12	CLIC4;	hsa-miR-421; hsa-miR-221-3p; hsa-miR-222-3p; hsa-miR-145-5p
SmoothMuscle	5.00E-12	WDR1;	hsa-miR-6741-5p
SmoothMuscle	5.00E-12	DOCK7;	hsa-miR-421; hsa-miR-214-5p; hsa-miR-197-5p
SmoothMuscle	5.00E-12	RND3;	hsa-miR-3679-5p
SmoothMuscle	5.00E-12	MRC2;	hsa-miR-184; hsa-miR-1909-3p; hsa-miR-23b-5p; hsa-miR-3679-5p
SmoothMuscle	5.00E-12	C1QTNF1;	hsa-miR-181a-5p
SmoothMuscle	5.00E-12	PGM3;	hsa-miR-214-5p
SmoothMuscle	5.00E-12	ITGAV;	hsa-miR-214-5p; hsa-miR-615-5p; hsa-miR-615-5p; hsa-miR-1275
SmoothMuscle	5.00E-12	EDIL3;	hsa-miR-615-5p
SmoothMuscle	5.00E-12	TGIF1;	hsa-miR-504-3p
SmoothMuscle	5.00E-12	RRAS2;	hsa-miR-1229-5p
SmoothMuscle	5.00E-12	AP3B1;	hsa-miR-143-3p
SmoothMuscle	5.00E-12	NAV1;	hsa-miR-3180-3p; hsa-miR-6870-5p; hsa-miR-6165; hsa-miR-574-3p; hsa-miR-4269; hsa-miR-3135b
SmoothMuscle	5.00E-12	OSMR;	hsa-miR-3135b
SmoothMuscle	5.00E-12	TGFBR2;	hsa-miR-933; hsa-miR-99a-5p
SmoothMuscle	5.00E-12	ENAH;	hsa-miR-874-3p
SmoothMuscle	5.00E-12	GREM1;	hsa-miR-4685-5p; hsa-miR-296-3p; hsa-miR-1224-5p; hsa-miR-1182
SmoothMuscle	5.00E-12	CTTN;	hsa-miR-501-3p; hsa-miR-421; hsa-miR-222-3p; hsa-miR-874-3p; hsa-miR-1909-3p; hsa-miR-557; hsa-miR-1224-5p; hsa-miR-214-5p; hsa-miR-615-5p
SmoothMuscle	5.00E-12	ASPH;	hsa-miR-146a-5p; hsa-miR-181a-5p; hsa-miR-328-3p
SmoothMuscle	5.00E-12	COL6A2;	hsa-miR-328-3p; hsa-miR-143-3p; hsa-miR-145-5p; hsa-miR-502-3p; hsa-miR-222-3p; hsa-miR-146a-5p; hsa-miR-214-5p; hsa-miR-296-3p; hsa-miR-1909-3p; hsa-miR-615-5p;
SmoothMuscle	5.00E-12	PXDN;	hsa-miR-3663-3p

SmoothMuscle	5.00E-12	CDH11;	hsa-miR-222-3p; hsa-miR-143-3p; hsa-miR-1224-5p; hsa-miR-502-3p; hsa-miR-222-3p; hsa-miR-145-5p; hsa-miR-328-3p; hsa-miR-221-3p; hsa-miR-421; hsa-miR-708-5p
SmoothMuscle	5.00E-12	PLIN3;	hsa-miR-23b-5p
SmoothMuscle	5.00E-12	ITGA5;	hsa-miR-214-5p; hsa-miR-1909-3p; hsa-miR-615-5p; hsa-miR-615-5p
SmoothMuscle	5.00E-12	BCAT1;	hsa-miR-143-3p; hsa-miR-145-5p; hsa-let-7i-5p
SmoothMuscle	5.00E-12	DBN1;	hsa-miR-4685-5p; hsa-miR-23b-5p; hsa-miR-3679-5p
SmoothMuscle	5.00E-12	RAB1A;	hsa-miR-6860; hsa-miR-1587; hsa-miR-6870-5p
SmoothMuscle	5.00E-12	RALA;	hsa-miR-197-5p
SmoothMuscle	5.00E-12	LRRC17;	hsa-miR-1909-3p
SmoothMuscle	5.00E-12	PTGS2;	hsa-miR-6870-5p
SmoothMuscle	5.00E-12	FSTL1;	hsa-miR-146a-5p; hsa-miR-1182; hsa-miR-502- 3p
SmoothMuscle	5.00E-12	THBS1;	hsa-miR-6742-5p; hsa-miR-6824-5p; hsa-miR-504-3p; hsa-miR-933
SmoothMuscle	5.00E-12	PTGS1;	hsa-miR-6846-5p; hsa-miR-4721; hsa-miR-574-3p
SmoothMuscle	5.00E-12	CD59;	hsa-miR-99a-5p; hsa-miR-143-3p; hsa-miR-34a-5p; hsa-miR-874-3p
SmoothMuscle	5.00E-12	PDLIM5;	hsa-miR-150-3p; hsa-miR-6870-5p
SmoothMuscle	5.00E-12	GPC6;	hsa-miR-557; hsa-miR-1275
SmoothMuscle	5.00E-12	CSNK1A1;	hsa-miR-421; hsa-miR-222-3p
SmoothMuscle	5.00E-12	GALNT2;	hsa-miR-214-5p
SmoothMuscle	5.00E-12	TXNRD1;	hsa-miR-6741-5p
SmoothMuscle	5.00E-12	YIPF5;	hsa-miR-7107-5p
SmoothMuscle	5.00E-12	LHFPL2;	hsa-miR-4269; hsa-miR-214-5p
SmoothMuscle	5.00E-12	GTF2H5;	hsa-miR-615-5p; hsa-miR-328-3p ; hsa-miR-214-5p
SmoothMuscle	5.00E-12	ARCN1;	hsa-miR-222-3p; hsa-miR-34a-5p; hsa-miR-99a-5p; hsa-miR-143-3p; hsa-miR-34a-5p; hsa-miR-874-3p
SmoothMuscle	5.00E-12	FKBP1A;	hsa-miR-328-3p; hsa-miR-1182
SmoothMuscle	5.00E-12	COL3A1;	hsa-miR-328-3p; hsa-miR-143-3p; hsa-miR-145-5p; hsa-miR-502-3p; hsa-miR-221-3p; hsa-miR-222-3p; hsa-miR-421; hsa-miR-708-5p; hsa-miR-146a-5p; hsa-miR-4685-5p; hsa-miR-296-3p; hsa-miR-1224-5p; hsa-miR-214-5p; hsa-miR-296-3p; hsa-miR-1909-3p; hsa-miR-3679-5p
SmoothMuscle	5.00E-12	MARCKS;	hsa-miR-574-3p; hsa-miR-184; hsa-miR-4685-5p; hsa-miR-1909-3p; hsa-miR-615-5p
SmoothMuscle	5.00E-12	MYO1B;	hsa-miR-4685-5p
SmoothMuscle	5.00E-12	KLHL5;	hsa-miR-214-5p; hsa-miR-1275; hsa-miR-328-3p
SmoothMuscle	5.00E-12	NFIB;	hsa-miR-1587 ; hsa-miR-3663-3p; hsa-miR-933; hsa-miR-214-5p; hsa-miR-150-3p
SmoothMuscle	5.00E-12	AXL;	hsa-miR-145-5p; hsa-miR-34a-5p; hsa-miR-222-3p; hsa-miR-143-3p; hsa-miR-1275
SmoothMuscle	5.00E-12	DLC1;	hsa-miR-99a-5p; hsa-miR-146a-5p
SmoothMuscle	5.00E-12	NEDD4;	hsa-miR-184
SmoothMuscle	5.00E-12	RAB13;	hsa-miR-574-3p
SmoothMuscle	5.00E-12	SEC24D;	hsa-miR-6846-5p
Endothelial cell:Muscle	0.002907	PECAM1;	hsa-miR-3157-3p; hsa-miR-3679-5p; hsa-miR-6846-5p
Endothelial cell:Muscle	0.002907	NCAM1;	hsa-miR-6870-5p; hsa-miR-184; hsa-miR-4269; hsa-miR-3135b

Endothelial cell:Muscle	0.002907	CD34	hsa-miR-181a-5p; hsa-let-7i-5p; hsa-miR-99a-5p; hsa-miR-143-3p; hsa-miR-34a-5p; hsa-miR-222-3p; hsa-miR-328-3p; hsa-miR-145-5p; hsa-miR-502-3p; hsa-miR-421; hsa-miR-708-5p; hsa-miR-557; hsa-miR-1224-5p; hsa-miR-214-5p; hsa-miR-296-3p; hsa-miR-1909-3p; hsa-miR-184; hsa-miR-3679-5p
Smooth Muscle cell:Blood Vessel	0.005261	COL15A1;	hsa-miR-1224-5p; hsa-miR-502-3p; hsa-miR-222-3p; hsa-miR-145-5p; hsa-miR-328-3p; hsa-miR-143-3p; hsa-miR-328-3p; hsa-miR-502-3p; hsa-miR-221-3p; hsa-miR-222-3p
Smooth Muscle cell:Blood Vessel	0.005261	TAGLN;	hsa-miR-6824-5p; hsa-miR-6742-5p
Smooth Muscle cell:Blood Vessel	0.005261	SPARC;	hsa-miR-574-3p
Smooth Muscle cell:Blood Vessel	0.005261	HSPB6;	hsa-miR-615-5p; hsa-miR-615-5p; hsa-miR-296-3; hsa-miR-874-3p
Smooth Muscle cell:Blood Vessel	0.005261	ELN;	hsa-miR-557; hsa-miR-1224-5p; hsa-miR-328-3p; hsa-miR-502-3p; hsa-miR-615-5p; hsa-miR-3679-5p
Smooth Muscle cell:Blood Vessel	0.005261	SYNPO2;	hsa-miR-6824-5p
Smooth Muscle cell:Blood Vessel	0.005261	HSPB7;	hsa-miR-615-5p; hsa-miR-296-3; hsa-miR-874-3p
Smooth Muscle cell:Blood Vessel	0.005261	LMOD1;	hsa-miR-1275; hsa-miR-296-3p
Smooth Muscle cell:Blood Vessel	0.005261	LRRC32;	hsa-miR-615-5p; hsa-miR-296-3p
Smooth Muscle cell:Blood Vessel	0.005261	THBS2;	hsa-miR-6742-5p
Smooth Muscle cell:Blood Vessel	0.005261	PCDH18;	hsa-miR-184
Smooth Muscle cell:Blood Vessel	0.005261	MYLK;	hsa-miR-34a-3p; hsa-miR-3135b
Smooth Muscle cell:Blood Vessel	0.005261	ADAMTS5;	hsa-miR-195-5p; hsa-miR-181a-5p; hsa-miR-222-3p; hsa-miR-30b-5p;hsa-miR-328-3p; hsa-miR-143-3p; hsa-miR-145-5p; hsa-miR-502-3p; hsa-miR-221-3p
Smooth Muscle cell:Blood Vessel	0.005261	FGF7;	hsa-miR-502-3p; hsa-miR-615-5p; hsa-miR-184; hsa-miR-3679-5p
Smooth Muscle cell:Blood Vessel	0.005261	ADAMTS2;	hsa-miR-30b-5p; hsa-miR-195-5p; hsa-miR-181a-5p; hsa-miR-222-3p; hsa-miR-30b-5p; hsa-miR-1224-5p; hsa-miR-502-3p; hsa-miR-222-3p; hsa-miR-145-5p; hsa-miR-328-3p; hsa-miR-143-3p;hsa-miR-502-3p; hsa-miR-221-3p; hsa-miR-557; hsa-miR-1224-5p
Smooth Muscle cell:Blood Vessel	0.005261	SGCD;	hsa-miR-23a-5p; hsa-miR-504-3p
Smooth Muscle cell:Blood Vessel	0.005261	SLIT3;	hsa-miR-6889-5p
Smooth Muscle cell:Blood Vessel	0.005261	PDGFRA;	hsa-miR-3157-3p
Smooth Muscle cell:Blood Vessel	0.005261	PRRX1;	hsa-miR-1587; hsa-miR-6870-5p; hsa-miR-150-3p
Smooth Muscle cell:Blood Vessel	0.005261	MMP2;	hsa-miR-6846-5p
Smooth Muscle cell:Blood Vessel	0.005261	ITGA1;	hsa-miR-214-5p; hsa-miR-1909-3p; hsa-miR-296-3p; hsa-miR-615-5p; hsa-miR-615-5p; hsa-miR-1275
Smooth Muscle cell:Blood Vessel	0.005261	BGN;	hsa-miR-181a-5p; hsa-miR-222-3p; hsa-miR-30b-5p ; hsa-miR-328-3p; hsa-miR-143-3p; hsa-miR-145-5p; hsa-miR-328-3p; hsa-miR-502-3p; hsa-miR-221-3p; hsa-miR-222-3p; hsa-miR-421; hsa-miR-708-5p; hsa-miR-557; hsa-miR-1224-5p; hsa-miR-146a-5p; hsa-miR-557; hsa-miR-1182; hsa-miR-502-3p
Smooth Muscle cell:Blood Vessel	0.005261	ADAM33;	hsa-miR-195-5p
Smooth Muscle cell:Blood Vessel	0.005261	ASPN;	hsa-miR-181a-5p; hsa-miR-328-3p; hsa-miR-143-3p; hsa-miR-145-5p; hsa-miR-502-3p; hsa-miR-221-3p
Smooth Muscle cell:Blood Vessel	0.005261	COL1A1;	hsa-miR-328-3p; hsa-miR-143-3p; hsa-miR-145-5p; hsa-miR-502-3p; hsa-miR-328-3p; hsa-miR-221-3p; hsa-miR-222-3p; hsa-miR-421; hsa-miR-708-5p; hsa-miR-557; hsa-miR-1224-5p; hsa-miR-146a-5p; hsa-miR-4685-5p; hsa-miR-296-3p; hsa-miR-1224-5p; hsa-miR-1182;hsa-miR-214-5p; hsa-miR-296-3p; hsa-miR-1909-3p
Smooth Muscle cell:Blood Vessel	0.005261	MFAP4;	hsa-miR-4685-5p; hsa-miR-1909-3p
Smooth Muscle cell:Blood Vessel	0.005261	COL3A1;	hsa-miR-328-3p; hsa-miR-143-3p; hsa-miR-145-5p; hsa-miR-502-3p; hsa-miR-221-3p; hsa-miR-421; hsa-miR-708-5p; hsa-miR-146a-5p; hsa-miR-4685-5p; hsa-miR-296-3p; hsa-miR-1224-5p; hsa-miR-214-5p; hsa-miR-296-3p; hsa-miR-1909-3p

Smooth Muscle cell:Blood Vessel	0.005261	COL1A2;	hsa-miR-328-3p; hsa-miR-143-3p; hsa-miR-145-5p; hsa-miR-502-3p; hsa-miR-502-3p; hsa-miR-221-3p; hsa-miR-222-3p; hsa-miR-421; hsa-miR-708-5p; hsa-miR-146a-5p; hsa-miR-1182; hsa-miR-214-5p; hsa-miR-296-3p; hsa-miR-1909-3p
Smooth Muscle cell:Blood Vessel	0.005261	CXCL12;	hsa-miR-421; hsa-miR-708-5p; hsa-miR-557; hsa-miR-1224-5p; hsa-miR-502-3p; hsa-miR-296-3p; hsa-miR-1909-3p; hsa-miR-615-5p; hsa-miR-184; hsa-miR-3679-5p
Smooth Muscle cell:Blood Vessel	0.005261	COL6A2;	hsa-miR-328-3p; hsa-miR-143-3p; hsa-miR-145-5p; hsa-miR-502-3p; hsa-miR-222-3p; hsa-miR-146a-5p; hsa-miR-296-3p; hsa-miR-1909-3p;
Smooth Muscle cell:Blood Vessel	0.005261	COL5A2;	hsa-miR-328-3p; hsa-miR-143-3p; hsa-miR-145-5p; hsa-miR-502-3p; hsa-miR-557; hsa-miR-1224-5p; hsa-miR-146a-5p; hsa-miR-214-5p; hsa-miR-296-3p; hsa-miR-1909-3p
Smooth Muscle cell:Blood Vessel	0.005261	CDH11;	hsa-miR-222-3p; hsa-miR-143-3p; hsa-miR-1224-5p; hsa-miR-502-3p; hsa-miR-222-3p; hsa-miR-145-5p; hsa-miR-328-3p; hsa-miR-221-3p; hsa-miR-421; hsa-miR-708-5p
Smooth Muscle cell:Blood Vessel	0.005261	RHOJ;	hsa-miR-6846-5p
Smooth Muscle cell:Blood Vessel	0.005261	ANGPTL2;	hsa-miR-30b-5p; hsa-miR-181a-5p; hsa-miR-222-3p; hsa-miR-30b-5p; hsa-miR-328-3p; hsa-miR-143-3p; hsa-miR-145-5p; hsa-miR-502-3p; hsa-miR-221-3p;hsa-miR-146a-5p; hsa-miR-296-3p; hsa-miR-1909-3p
Smooth Muscle cell:Blood Vessel	0.005261	FBN1	hsa-miR-502-3p; hsa-miR-146a-5p; hsa-miR-1909-3p; hsa-miR-296-3p; hsa-miR-615-5p
